# Acupuncture vs Massage for Pain in Patients Living With Advanced Cancer: The IMPACT Randomized Clinical Trial

**DOI:** 10.1001/jamanetworkopen.2023.42482

**Published:** 2023-11-14

**Authors:** Andrew S. Epstein, Kevin T. Liou, Sally A. D. Romero, Raymond E. Baser, Greta Wong, Han Xiao, Zunli Mo, Desiree Walker, Jodi MacLeod, Qing Li, Margaret Barton-Burke, Gary E. Deng, Katherine S. Panageas, John T. Farrar, Jun J. Mao

**Affiliations:** 1 Department of Medicine, Memorial Sloan Kettering Cancer Center, New York, New York; 2 Department of Obstetrics, Gynecology, and Reproductive Sciences, University of California, San Diego; 3 Department of Epidemiology and Biostatistics, Memorial Sloan Kettering Cancer Center, New York, New York; 4 Cancer Patient Support Center, Baptist Health Miami Cancer Institute, Miami, Florida; 5 Office of Nursing Research, Memorial Sloan Kettering Cancer Center, New York, New York; 6 Department of Biostatistics and Epidemiology, Perelman School of Medicine, University of Pennsylvania, Philadelphia

## Abstract

**Importance:**

Pain is challenging for patients with advanced cancer. While recent guidelines recommend acupuncture and massage for cancer pain, their comparative effectiveness is unknown.

**Objective:**

To compare the effects of acupuncture and massage on musculoskeletal pain among patients with advanced cancer.

**Design, Setting, and Participants:**

A multicenter pragmatic randomized clinical trial was conducted at US cancer care centers consisting of a northeastern comprehensive cancer center and a southeastern cancer institute from September 19, 2019, through February 23, 2022. The principal investigator and study statisticians were blinded to treatment assignments. The duration of follow-up was 26 weeks. Intention-to-treat analyses were performed (linear mixed models). Participants included patients with advanced cancer with moderate to severe pain and clinician-estimated life expectancy of 6 months or more. Patient recruitment strategy was multipronged (eg, patient database queries, mailings, referrals, community outreach). Eligible patients had English or Spanish as their first language, were older than 18 years, and had a Karnofsky score greater than or equal to 60 (range, 0-100; higher scores indicating less functional impairment).

**Interventions:**

Weekly acupuncture or massage for 10 weeks with monthly booster sessions up to 26 weeks.

**Main Outcomes and Measures:**

The primary end point was the change in worst pain intensity score from baseline to 26 weeks. The secondary outcomes included fatigue, insomnia, and quality of life. The Brief Pain Inventory (range, 0-10; higher numbers indicate worse pain intensity or interference) was used to measure the primary outcome. The secondary outcomes included fatigue, insomnia, and quality of life.

**Results:**

A total of 298 participants were enrolled (mean [SD] age, 58.7 [14.1] years, 200 [67.1%] were women, 33 [11.1%] Black, 220 [74.1%] White, 46 [15.4%] Hispanic, and 78.5% with solid tumors). The mean (SD) baseline worst pain score was 6.9 (1.5). During 26 weeks, acupuncture reduced the worst pain score, with a mean change of −2.53 (95% CI, −2.92 to −2.15) points, and massage reduced the Brief Pain Inventory worst pain score, with a mean change of −3.01 (95% CI, −3.38 to −2.63) points; the between-group difference was not significant (−0.48; 95% CI, −0.98 to 0.03; *P* = .07). Both treatments also improved fatigue, insomnia, and quality of life without significant between-group differences. Adverse events were mild and included bruising (6.5% of patients receiving acupuncture) and transient soreness (15.1% patients receiving massage).

**Conclusions and Relevance:**

In this randomized clinical trial among patients with advanced cancer, both acupuncture and massage were associated with pain reduction and improved fatigue, insomnia, and quality of life over 26 weeks; however, there was no significant different between the treatments. More research is needed to evaluate how best to integrate these approaches into pain treatment to optimize symptom management for the growing population of people living with advanced cancer.

**Trial Registration:**

ClinicalTrials.gov Identifier: NCT04095234

## Key Points

**Question:**

What is the effectiveness of acupuncture and massage for cancer pain in patients with advanced cancer?

**Findings:**

In this randomized clinical trial that included 298 patients with advanced cancer, both acupuncture and massage were associated with long-term pain reduction. There was no significant difference between treatments.

**Meaning:**

The findings of this study suggest that both acupuncture and massage may offer pain relief as integrative modalities in patients with advanced cancer.

## Teaser

This randomized clinical trial compares the effectiveness of acupuncture and massage as treatment for musculoskeletal pain in patients with advanced cancer.

## Introduction

Pain is a debilitating symptom that affects up to two-thirds of patients with advanced cancer.^1,2^ Despite its prevalence in this population, pain remains and often occurs concomitantly with fatigue and insomnia, presenting additional treatment challenges.^3,4,5,6,7,8,9^ Oncologic pain management has historically relied on drug therapies; however, with the ongoing opioid crisis, many health care professionals are hesitant to prescribe opioids, while some patients with cancer see their legitimate access to pain medication curtailed.^10^ Furthermore, many patients are concerned about adverse effects of opioids and other medications, which leads to undertreated pain and preference for nonpharmacologic pain treatments.^11^

In 2022, the American Society of Clinical Oncology and the Society for Integrative Oncology published a joint guideline recommending acupuncture and massage be considered for cancer pain management.^12^ Acupuncture has demonstrated long-term effectiveness for joint pain related to aromatase inhibitors among breast cancer survivors^13^ and for musculoskeletal pain among survivors of various cancer types^14^; however, there is a paucity of research among patients with advanced cancer.^15^ Massage is more effective than simple touch for short-term pain reduction in patients with cancer receiving hospice care,^16^ but previous trials often have short follow-up duration. The benefit of massage remains uncertain as related to other interventions such as acupuncture.^17^

With improvements in treatment, people are living longer with advanced cancer.^18^ Therefore, managing pain and comorbid symptoms is critical for quality of life in this growing population. Although acupuncture and massage have recently been recommended for treatment of cancer pain, to our knowledge, no studies have compared their effectiveness in advanced cancer populations. Furthermore, most randomized trials on pain interventions in patients with advanced cancer had only a short intervention or follow-up.^19,20^ To inform patients and health care professionals with evidence on how to make decisions on incorporating nonpharmacologic interventions for pain and symptom management, we conducted a randomized clinical trial to evaluate the long-term comparative effectiveness of acupuncture vs massage for pain and comorbid fatigue and insomnia in patients living with advanced cancer.

## Methods

### Trial Oversight

The Integrative Medicine for Pain in Patients with Advanced Cancer Trial (IMPACT) is a pragmatic, 2-arm, parallel-group randomized clinical trial. The study (Supplement 1) was approved by the institutional review board at Memorial Sloan Kettering Cancer Center and has been previously published.^21^ All participants gave written informed consent; financial compensation was provided. This study followed the Consolidated Standards of Reporting Trials (CONSORT) reporting guideline.

### Patients

To ensure participant diversity, our recruitment strategy included patient database queries, letter mailings, clinician referrals, and community outreach and engagement. Recruitment sites included Memorial Sloan Kettering Cancer Center, a National Cancer Institute–Designated Comprehensive Cancer Center, with a main campus in Manhattan and regional sites in New York (Westchester County, Long Island), New Jersey (Bergen, Monmouth, and Basking Ridge), and Florida (Baptist Health Miami Cancer Institute).

Patients were eligible if they were fluent in English or Spanish, they were older than 18 years, had a Karnofsky score greater than or equal to 60 (range, 0-100; with score of 60 indicating unable to work, able to live at home and care for most personal needs, with varying amount of assistance needed),^22^ and had an advanced cancer diagnosis. Advanced cancer was defined as follows: stage III or IV lung cancer, any stage pancreatic cancer, unresectable cholangiocarcinoma, unresectable liver cancer, unresectable ampullary or periampullary cancer or other stage IV gastrointestinal cancer, stage III or IV ovarian or fallopian tube cancer or other stage IV gynecologic cancer, stage IV breast cancer, stage IV genitourinary cancer, stage III or IV sarcoma, stage IV melanoma, stage III or IV head/neck cancer, stage IV endocrine cancer, or hematologic malignant neoplasms (lymphoma, myeloma, and leukemia). Patients were eligible only if they were deemed by a clinician to have an expected prognosis of 6 or more months.

To be eligible, patients must have also reported musculoskeletal pain, defined as regional (eg, specific joints) or generalized (ie, fibromyalgia), as the primary source of pain. The pain must have been present for at least 1 month and for at least 15 of the preceding 30 days. Patients must also have rated their worst pain intensity as 4 or greater on a numerical rating scale of 0 to 10. Patients were excluded if they had a platelet count less than 150 × 10^3^/μL (to convert to ×10^9^ per liter, multiply by 1).

### Trial Procedures

Potential participants were screened by a research coordinator, and a study clinician confirmed their eligibility. After providing informed consent, participants completed baseline assessments and underwent randomization. Race and ethnicity data (from patients’ self-reports) were included to identify demographic characteristics associated with pain reduction from either intervention. Patients were randomized to acupuncture or massage in a 1:1 ratio on a secure computer system that ensured full allocation concealment and used permuted blocks stratified by baseline opioid use (yes vs no) and by accrual sites. The principal investigator (J.J.M.) and study statisticians (R.E.B. and K.S.P.) were blinded to treatment assignments. Participants completed patient-reported outcomes using Research Electronic Data Capture at weeks 0, 4, 10, 14, 18, 22, and 26.

### Assessments and Outcomes

The primary outcome was worst pain severity in the past week with response choices ranging from 0 (no pain) to 10 (pain as bad as you can imagine) measured by the short-form Brief Pain Inventory (BPI). The BPI is a reliable, valid, and responsive measure of pain (Cronbach α = 0.77-0.91)^23^ containing 4 pain severity items and 7 pain interference items rated on a scale from 0 to 10, with higher numbers indicating worse pain intensity or interference. The individual clinical response benchmark for BPI worst pain is a 30% improvement from baseline.^24^ To assess comorbid symptoms and health-related quality of life, we used the Brief Fatigue Inventory,^25^ the Insomnia Severity Index,^26^ and the Patient-Reported Outcomes Measurement Information System Scale, version 1.2 (Global Health).^27^ We tracked use of analgesic medications (eg, acetaminophen, nonsteroidal anti-inflammatory drugs, opioids, and adjuvants for neuropathic pain) using weekly pain medication diaries. To monitor treatment safety, study therapists and research coordinators collected data on adverse events using the Common Terminology Criteria for Adverse Events, version 5.^28^

### Interventions

Licensed and oncology-experienced acupuncturists and massage therapists delivered the treatments. All therapists received a manual with the treatment protocols for acupuncture and massage and were trained by the principal investigator (J.J.M.), lead acupuncturist, and/or lead massage therapist. The lead therapists audited at least 2 records for each therapist per week to ensure treatment fidelity. If treatments needed to be modified for medical reasons, therapists were instructed to document the details of the modifications with an accompanying rationale. The interventions have been described in detail previously.^21^

Acupuncturists placed 10 to 20 needles at a minimum of 4 local points around the body area with the most pain, as well as supplementary points at other locations, depending on the presence of comorbid symptoms. The acupuncture needles were inserted to appropriate depths based on the body type and point location.^14^ The acupuncturist manipulated needles to achieve de qi, a local sensation of soreness or distension that accompanies effective needling.^29^ The needles at the 4 local points for pain were electrically stimulated at 2 Hz using a transcutaneous electrical nerve stimulation unit. For participants with electronically charged medical devices, no electrical stimulation was used. Total treatment time was 30 minutes per session with needles left in place for 20 minutes.

Massage therapists started with a 5-minute protocol that included guided diaphragmatic breathing exercise, rib mobilizations, and occipital release designed to increase parasympathetic tone. Depending on the primary area of pain, the therapist focused 20 minutes of massage on that specific body area followed by effleurage toward the heart. Massage techniques were applied with light to moderate pressure and included compression, muscle stripping, active/passive range of motion, postisometric stretching, effleurage, myofascial release, positional release, and trigger point release for a total treatment time of 30 minutes.^30,31^

### Statistical Analysis

All randomized patients were included in the analysis using the intention-to-treat principle. For the primary outcome, we used a prespecified linear mixed model in which we constrained the treatment arms to have a common baseline mean,^32^ reflecting the prerandomization timing of the baseline assessment. The dependent variable vector included the baseline (week 0) BPI worst pain and all postrandomization assessments at weeks 4, 10, 14, 18, 22, and 26. The independent variables were the randomization stratification variables (accrual site and baseline opioid use), treatment arm, week (categorical), and the arm-by-week interaction. A patient-level random intercept was included in the model to account for the repeated outcome measurements within patients. Linear mixed models provide valid inference under the reasonable assumption that missing follow-up data were missing at random.^33^ We therefore did not impute missing values. Results are reported as least square means, mean differences, and 95% CIs, with inferences regarding differences between arms based on model coefficients from the arm-by-week interaction. We prespecified comparisons between arms at 2 time points of interest: weeks 10 and 26. Differences in week 26 BPI worst pain was the primary end point of the study, allowing for evaluation of long-term treatment effects. Week 10 BPI worst pain was a secondary end point to evaluate the treatment effect at the end of treatment. We used similar models to analyze our continuous secondary outcomes. To analyze pain medication use (dichotomous), we used a generalized linear mixed model with logistic link function and with the arms constrained to have a common baseline probability of analgesic medication use. The analgesic medication use model was otherwise specified similarly to the models for the continuous outcome measures. We did not adjust the CIs for multiple testing of secondary outcomes. Because of the potential for inflated type I error due to multiple comparisons, results from analyses of secondary outcomes were interpreted as exploratory. We also conducted additional sensitivity analyses to evaluate whether pain medication use or COVID-19 treatment interruption had any influence on our primary outcome (eMethods and the eTable in Supplement 2). All analyses were performed in R, version 4.2.2 (R Foundation for Statistical Computing).

We calculated that with a sample size of 300, assuming 20% loss to follow-up by 26 weeks, Pearson correlation between baseline and 26-week BPI worst pain of 0.5 and 2-sided α of .05 would provide the trial with 80% power to detect a Cohen *d* value of 0.35. Because this is a comparison between 2 interventions, it was unpaired. Based on our preliminary data in patients with advanced cancer who experienced moderate to severe pain (n = 284), the BPI worst pain score had an SD of 1.7. A 1-point difference is considered a minimal clinically meaningful difference in pain research.^34^ Given that a difference of 1 on the BPI worst pain scale, based on SD of 1.7, equals a Cohen *d* value of 0.59, the trial was sufficiently powered to detect a clinically meaningful difference between acupuncture and massage at 26 weeks.

## Results

### Patients

**Figure 1.**
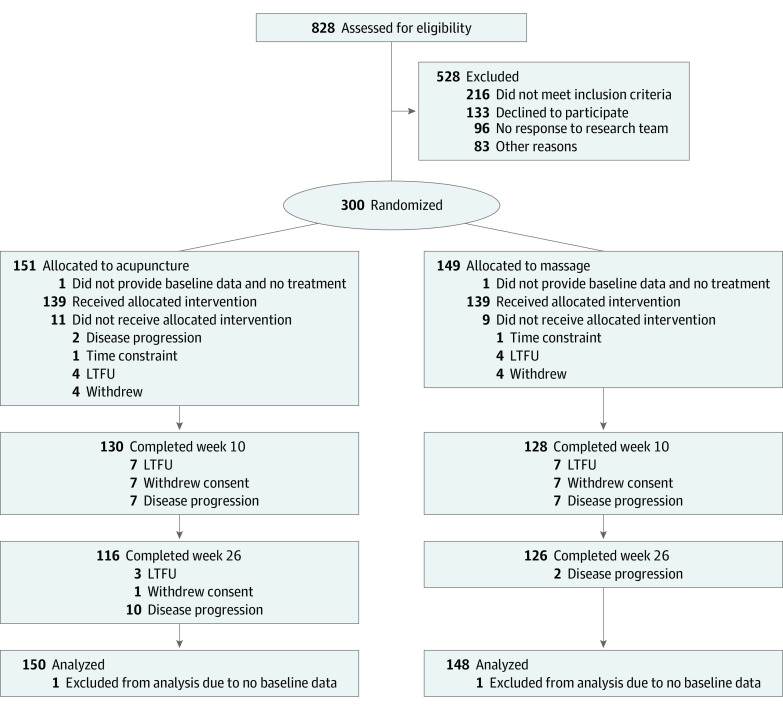
Trial Enrollment and Follow-Up LTFU indicates lost to follow-up.

From September 19, 2019, through February 23, 2022, we screened 828 patients, and 528 were excluded due to ineligibility or unwillingness to participate. Of the 300 enrolled patients, 151 were randomly assigned to acupuncture and 149 to massage. Two patients did not provide baseline data and did not receive treatment; therefore, these 2 patients were excluded from the analyses. Among 150 individuals in the acupuncture group, 139 (92.7%) received at least 1 treatment and 92 (61.3%) completed 10 or more treatments. Among 148 individuals in the massage group, 139 (93.9%) received at least 1 treatment, and 99 (66.9%) completed 10 or more treatments. Among all participants, 56 (18.8%) withdrew from data collection at the week 26 primary end point (Figure 1).

The demographic and clinical characteristics of the patients in the 2 groups were similar at baseline. The mean (SD) age of the patients was 58.7 (14.1) years, 200 (67.1%) were women, and 98 (32.9%) were men. Self-reported race and ethnicity, as categorized herein, were included to ensure diversity among research participants: 19 individuals (6.4%) were Asian, 33 (11.1%) were Black, 46 (15.4%) were Hispanic, 220 (74.1%) were White, and 25 (8.4%) were multiracial.

A total of 78.5% of the patients had solid tumors. The most common cancer types were hematologic (21.5%), breast (19.8%), gynecologic (14.4%), and gastrointestinal (11.7%). Mean (SD) time since diagnosis was 5.6 (7.5) years. The mean (SD) score for worst pain severity was 6.9 (1.5), the mean (SD) pain duration was 3.8 (7.3) years, and 98 individuals (32.9%) were receiving opioids at baseline (Table 1).

**Table 1.** Baseline Characteristics of Participants

Characteristic	No. (%)		
	Total (n = 298)	Acupuncture (n = 150)	Massage (n = 148)
Age, mean (SD), y	58.7 (14.1)	58.4 (14.4)	58.9 (13.9)
Sex			
Female	200 (67.1)	104 (69.3)	96 (64.9)
Male	98 (32.9)	46 (30.7)	52 (35.1)
Race			
Asian	19 (6.4)	8 (5.4)	11 (7.4)
Black	33 (11.1)	18 (12.1)	15 (10.1)
White	220 (74.1)	111 (74.5)	109 (73.7)
Multiracial	25 (8.4)	12 (8.1)	13 (8.8)
Ethnicity			
Hispanic	46 (15.4)	21 (14.0)	25 (16.9)
Non-Hispanic	252 (84.6)	129 (86.0)	123 (83.1)
Cancer type			
Breast	59 (19.8)	28 (18.7)	31 (20.9)
Lung	29 (9.7)	15 (10.0)	14 (9.5)
Prostate	29 (9.7)	13 (8.7)	16 (10.8)
Gynecologic	43 (14.4)	27 (18.0)	16 (10.8)
Gastrointestinal	35 (11.7)	16 (10.7)	19 (12.8)
Hematologic	64 (21.5)	35 (23.3)	29 (19.6)
Head and neck	18 (6.0)	9 (6.0)	9 (6.1)
Other	21 (7.1)	7 (4.7)	14 (9.5)
Cancer treatments			
Surgery	187 (62.8)	87 (58.0)	100 (67.6)
Chemotherapy	250 (83.9)	127 (84.7)	123 (83.1)
Radiotherapy	163 (54.7)	76 (50.7)	87 (58.8)
Immunotherapy/biological therapy	87 (29.2)	36 (24.0)	51 (34.5)
Hormonal	75 (25.2)	36 (24.0)	39 (26.4)
Years since cancer diagnosis, mean (SD), y	5.6 (7.5)	5.7 (7.9)	5.4 (7.0)
Duration of pain symptom, mean (SD), y^a^	3.8 (7.3)	3.1 (5.3)	4.6 (8.8)
Baseline measures			
Brief Pain Inventory severity, mean (SD)			
Worst pain item	6.9 (1.5)	6.9 (1.6)	6.9 (1.5)
Average pain item	5.4 (1.7)	5.4 (1.7)	5.5 (1.7)
Brief Pain Inventory interference, mean (SD)^b^	4.8 (2.2)	4.8 (2.1)	4.7 (2.3)
Brief Fatigue score, mean (SD)^c^	4.8 (2.4)	4.8 (2.2)	4.8 (2.5)
Insomnia score, mean (SD)^d^	13.6 (6.6)	13.4 (6.4)	13.7 (6.9)
PROMIS Global Health, mean (SD)			
Global Physical Health t score^e^	37.8 (6.7)	37.9 (6.6)	37.8 (6.8)
Global Mental Health t score^f^	42.6 (8.9)	42.9 (8.6)	42.3 (9.2)
Opioid use	98 (32.9)	50 (33.3)	48 (32.4)

Abbreviation: PROMIS, Patient-Reported Outcomes Measurement Information System.

^a^ Duration of pain symptoms reported by the patient and verified by clinicians before enrollment.

^b^ The Brief Pain Inventory interference score (range, 0-10) was the mean of the 7 interference items.

^c^ The Brief Fatigue score (range, 0-10) was the mean of the 9 fatigue items.

^d^ Insomnia score (range, 0-28) was the sum of the 7 items in the Insomnia Severity Index.

^e^ Global Physical Health t score (range, 16.2-67.7) is calculated by summing the 4 physical health items in the PROMIS scale and then converting to t scores.

^f^ Global Mental Health t score (range, 21.2-67.6) is calculated by summing the 4 mental health items in the PROMIS scale and then converting to t scores.

### Primary Outcome

**Figure 2.**
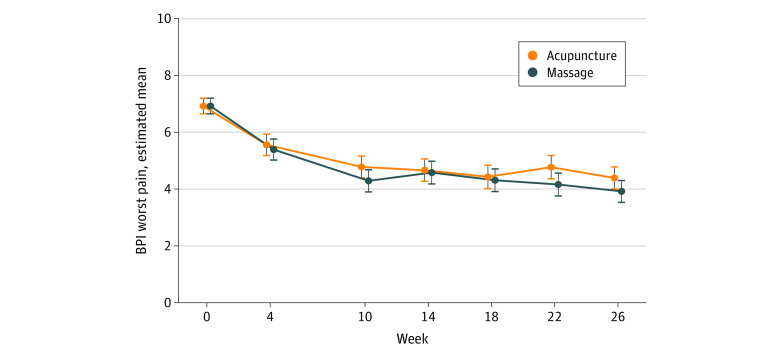
Estimated Brief Pain Inventory (BPI) Worst Pain Means by Week and Arm The BPI worst pain scores range from 0 to 10, with higher scores indicating worse pain. Data points represent the model-estimated BPI worst pain means and 95% CI (error bars) from a linear mixed model with baseline means constrained to be equal across study arms. The dependent variable vector included the prerandomization baseline (week 0) assessment, as well as all postrandomization assessments. The independent variables were the randomization stratification variables (accrual site and baseline opioid use), treatment arm, week (categorical), and the arm-by-week interaction. A patient-level random intercept was included in the model to account for the repeated outcome measurements within patients.

From baseline to week 26, acupuncture reduced the BPI worst pain score, with a mean change of −2.53 (95% CI, −2.92 to −2.15) points, and massage reduced the BPI worst pain score, with a mean change of −3.01 (95% CI, −3.38 to −2.63) points. The between-group difference in BPI worst pain reduction at week 26 was not significant (−0.48; 95% CI, −0.98 to 0.03; Cohen *d* = 0.31; *P* = .07) (Figure 2 and Table 2). More than half of the patients had a clinical response to treatment by week 26 (55.2%; 95% CI, 46.0%-64.0% for acupuncture; 65.9%; 95% CI, 57%-74% for massage).

**Table 2.** Estimates of Within-Arm and Between-Arm Differences for Primary and Secondary Outcomes^a^

Outcome measure	Acupuncture		Massage		Mean between-arm difference (95% CI)	*P* value	Effect size (95% CI)^b^
	Mean (95% CI)	Mean change (95% CI)	Mean (95% CI)	Mean change (95% CI)			
BPI worst pain							
At baseline	6.92 (6.65 to 7.20)	NA	6.92 (6.65 to 7.20)	NA	NA	NA	NA
At 10 wk	4.78 (4.40 to 5.16)	−2.14 (−2.52 to −1.77)	4.29 (3.90 to 4.68)	−2.63 (−3.02 to −2.25)	−0.48 (−0.99 to 0.02)	.06	−0.31 (−0.64 to 0.02)
At 26 wk	4.39 (4.00 to 4.78)	−2.53 (−2.92 to −2.15)	3.92 (3.53 to 4.30)	−3.01 (−3.38 to −2.63)	−0.48 (−0.98 to 0.03)	.07	−0.31 (−0.64 to 0.02)
Brief Fatigue Inventory							
At baseline	4.75 (4.48 to 5.02)	NA	4.75 (4.48 to 5.02)	NA	NA	NA	NA
At 10 wk	3.72 (3.35 to 4.09)	−1.03 (−1.38 to −0.69)	3.57 (3.19 to 3.94)	−1.18 (−1.53 to −0.84)	−0.15 (−0.62 to 0.32)	.53	−0.06 (−0.26 to 0.13)
At 26 wk	3.60 (3.22 to 3.99)	−1.15 (−1.50 to −0.79)	3.38 (3.01 to 3.75)	−1.37 (−1.72 to −1.03)	−0.22 (−0.70 to 0.25)	.35	−0.09 (−0.29 to 0.11)
Insomnia Severity Index							
At baseline	13.54 (12.79 to 14.29)	NA	13.54 (12.79 to 14.29)	NA	NA	NA	NA
At 10 wk	10.51 (9.51 to 11.51)	−3.03 (−3.93 to −2.13)	10.60 (9.59 to 11.62)	−2.94 (−3.85 to −2.02)	0.10 (−1.14 to 1.33)	.88	0.01 (−0.17 to 0.20)
At 26 wk	10.06 (9.03 to 11.09)	−3.49 (−4.42 to −2.56)	10.08 (9.07 to 11.08)	−3.46 (−4.37 to −2.56)	0.02 (−1.23 to 1.27)	.97	0.00 (−0.19 to 0.19)
PROMIS-GH Physical Health score							
At baseline	37.91 (37.10 to 38.71)	NA	37.91 (37.10 to 38.71)	NA	NA	NA	NA
At 10 wk	41.06 (40.00 to 42.12)	3.15 (2.22 to 4.07)	41.03 (39.96 to 42.10)	3.12 (2.18 to 4.06)	−0.03 (−1.30 to 1.24)	.96	−0.00 (−0.19 to 0.19)
At 26 wk	41.67 (40.58 to 42.75)	3.76 (2.80 to 4.71)	41.57 (40.51 to 42.63)	3.66 (2.73 to 4.59)	−0.10 (−1.39 to 1.19)	.88	−0.01 (−0.21 to 0.18)
PROMIS-GH Mental Health score							
At baseline	42.66 (41.64 to 43.67)	NA	42.66 (41.64 to 43.67)	NA	NA	NA	NA
At 10 wk	43.52 (42.23 to 44.82)	0.86 (−0.21 to 1.94)	44.02 (42.72 to 45.33)	1.36 (0.27 to 2.46)	0.50 (−0.99 to 1.99)	.51	0.06 (−0.11 to 0.22)
At 26 wk	44.75 (43.42 to 46.07)	2.09 (0.97 to 3.20)	44.65 (43.36 to 45.95)	2.00 (0.92 to 3.07)	−0.09 (−1.60 to 1.42)	.90	−0.01 (−0.18 to 0.16)

Abbreviations: BPI, Brief Pain Inventory; NA, not applicable; PROMIS-GH, Patient-Reported Outcomes Measurement Information System–Global Health.

^a^ For each outcome, estimates are derived from a linear mixed model with baseline means constrained to be equal across study arms. The dependent variable vector included the prerandomization baseline (week 0) assessment, as well as all postrandomization assessments (for BPI worst pain, at weeks 4, 10, 14, 18, 22, and 24, and for all other outcomes at weeks 10, 18, and 26). The independent variables were the randomization stratification variables (accrual site and baseline opioid use), treatment arm, week (categorical), and the arm-by-week interaction. A patient-level random intercept was included in the model to account for the repeated outcome measurements within patients.

^b^ Calculated as the mean between-arm difference divided by the pooled SD of the baseline scores.

### Secondary Outcomes

Both acupuncture and massage improved pain-related functional interference, fatigue, insomnia, and physical quality of life at week 26 relative to baseline (Table 2). The proportion of patients using pain medications at baseline was 54.7% (95% CI, 40.6%-68.1%), which decreased at week 26 to 27.5% (95% CI, 14.1%-46.7%) in the acupuncture arm and to 35.6% (95% CI, 19.7%-55.4%) in the massage arm.

### Adverse Events

Adverse events were mostly mild. Among patients receiving acupuncture, bruising (6.5%), localized pain (5.8%), and bleeding (1.4%) were the most commonly reported adverse events. Among patients receiving massage, transient soreness (15.1%) and headache (1.4%) were the most commonly reported adverse events. There was no documented transmission of COVID-19 during the receipt of the study interventions.

## Discussion

In this randomized clinical trial comparing acupuncture and massage, both therapies were associated with pain reduction and improved fatigue, insomnia, and quality of life over 26 weeks; however, there were no significant differences for pain or secondary outcomes. More than half of the participants had a clinically meaningful response to treatment. Both therapies were delivered safely during the COVID-19 pandemic with only mild adverse events. The findings contribute to current guidelines for cancer pain management by demonstrating the long-term comparative effectiveness of 2 nonpharmacologic therapies in the growing population of patients living with advanced cancer.^12,18^

The durable effects of acupuncture observed in this study are consistent with findings from other large randomized clinical trials of acupuncture for pain in the general population^35,36^ and cancer survivors with chronic pain.^13,14^ Consistent with prior research of pain in patients with cancer,^37^ acupuncture also reduced fatigue and insomnia, highlighting its capacity to jointly address multiple, co-occurring symptoms in a cancer population with a high symptom burden.

Past oncology massage research has demonstrated short-term benefits for pain, mood, and quality of life^38^ in patients with cancer receiving hospice care, but the improvements were not durable.^16^ Our study found massage was associated with reduced pain in both the short and long term; however, our intervention included booster treatments, monthly sessions for 4 months after the weekly sessions for 10 weeks, to consolidate the initial treatment effect. It is also possible that population differences may contribute to the observed differences, as our study enrolled patients living with advanced cancer rather than patients nearing the end of life. There may even be a small benefit of massage over acupuncture, albeit nonsignificant. The lack of difference between massage and acupuncture at week 26 confirmed that a course of massage therapy with booster sessions was likely to result in sustained pain reduction as previously reported in acupuncture studies.^14,39^

In patients with advanced cancer, pharmacotherapy is often the mainstay of pain management. However, polypharmacy is a growing concern in this population due to adverse effects and drug-drug interactions.^40,41^ In this trial, acupuncture and massage not only reduced pain but also improved comorbid fatigue and insomnia symptoms, underscoring the multifaceted benefits that integrative modalities can offer for this population. In this trial, interventions were provided in addition to pharmacotherapy for some patients, reflecting the clinical setting; therefore, our data should not be interpreted as drugs should be replaced by acupuncture or massage, but that these nonpharmacologic interventions can improve pain and symptom control while potentially reducing medication use.

Both acupuncture and massage are popular integrative medicine approaches that are increasingly available in both academic and community cancer centers.^42,43^ Currently, Medicare only covers acupuncture for chronic low back pain and does not cover massage for pain.^44^ Given that patients with advanced cancer often have pain in multiple locations due to their disease and oncologic treatment, expanding Medicare coverage to include other pain locations, as well as massage, is needed to promote equitable and effective pain management for patients with cancer. More educational effort should also be directed at training acupuncturists or massage therapists on safe and effective practice for patients with advanced cancer. Furthermore, more research is needed to understand how best to integrate these nonpharmacologic treatments into the current pain management strategy to create patient-centered, efficient, and effective care.

### Limitations

This trial has limitations. First, the study was designed as a pragmatic comparative effectiveness trial of 2 active interventions, so sham or usual care control groups were not used. Although both therapies have been shown to be superior to sham and usual care in prior research,^13,14,15,16,17,35^ the absence of a control arm in this study limits the capacity to interpret the extent to which the reduction in pain from baseline is attributed to the intervention and how much is due to a placebo effect. Second, patients and clinicians could not be blinded due to the nature of the interventions; however, the principal investigator and statisticians were blinded. Third, massage therapists had more patient-clinician contact time in the process of therapy delivery, but the treatment duration of 30 minutes was identical in both treatment groups. Fourth, it is possible that some of the improvement in pain may have been due to analgesic medication titration and/or tumor response to cancer therapy. However, controlling for medication use in sensitivity analyses did not impact our results or conclusions. In addition, while we longitudinally collected data (clinician progress notes, staging scan reports) on tumor status (progression, regression, stability), we did not perform independent review of adherence to cancer therapies or apply radiographic response evaluation criteria in solid tumors. Fifth, COVID-19 interrupted treatment for some patients; however, our results reflect the community setting effects of these treatments in the context of a pandemic. In additional sensitivity analyses, controlling for COVID-19–related treatment interruption did not change our results. Sixth, our therapists underwent rigorous training on safety and intervention delivery and had consistent fidelity monitoring; therefore, the results may not be generalizable in community settings, where there is wider variability in clinical care delivery.

## Conclusions

In this randomized clinical trial, both acupuncture and massage were associated with reduction in pain and improved fatigue, insomnia, and quality of life among patients living with advanced cancer during 26 weeks. However, there was no significant difference between treatments.

## Notes

Supplement 1.
Trial Protocol and Statistical Analysis Plan

Supplement 2. **eMethods.** Sensitivity Analyses Adjusting for COVID-19–Related Treatment Interruptions **eTable.** Estimates of Within-Arm and Between-Arm Differences for BPI Worst Pain, Controlling for COVID-19–Related Treatment Interruptions

Supplement 3.
Data Sharing Statement
