# Supplementary material for: Acupuncture vs Massage for Pain in Patients Living With Advanced Cancer: The IMPACT Randomized Clinical Trial
Source: JAMA Netw Open. 2023 Nov 14;6(11):e2342482. doi: 10.1001/jamanetworkopen.2023.42482 (PMC10646731; doi:10.1001/jamanetworkopen.2023.42482)
Supplement: Supplement 1. — Trial Protocol and Statistical Analysis Plan [file jamanetwopen-e2342482-s001.pdf]

MSK PROTOCOL COVER SHEET

Integrative Medicine for Pain in Patients with Advanced Cancer Trial (IMPACT)

Principal Investigator/Department:

Dr. Jun Mao, Integrative Medicine Service

Memorial Sloan Kettering Cancer Center
1275 York Avenue
New York, New York 10065

Table of Contents

1.0 PROTOCOL SUMMARY AND/OR SCHEMA 3
2.0 OBJECTIVES AND SCIENTIFIC AIMS 4
3.0 BACKGROUND AND RATIONALE 4
4.0 OVERVIEW OF STUDY DESIGN/INTERVENTION 9
4.1 Design 9
4.2 Intervention 9
5.0 THERAPEUTIC/DIAGNOSTIC AGENTS & NON-THERAPEUTIC ASSESSMENTS 11
6.0 CRITERIAFOR PARTICIPANT ELIGIBILITY 11
6.1 Participant Inclusion Criteria 11
6.2 Participant Exclusion Criteria 12
7.0 RECRUITMENT PLAN 12
7.1 Research Participant Registration 13
7.2 Randomization 13
8.0 INFORMED CONSENT PROCEDURES 14
9.0 PRE-TREATMENT/INTERVENTION 14
10.0 TREATMENT/INTERVENTION PLAN 16
11.0 EVALUATION DURING TREATMENT/INTERVENTION 16
12.0 CRITERIAFOR REMOVAL FROM STUDY 18
13.0 CRITERIAFOR OUTCOME ASSESSMENT AND ENDPOINT EVALUABILITY 19
14.0 BIOSTATISTICS 19
15.0 TOXICITIES/RISKS/SIDE EFFECTS 22
15.1 Serious Adverse Event (SAE) Reporting 23
15.2. External SAE Reporting 24
16.0 PROTECTION OF HUMAN PARTICIPANTS 24
16.1 Privacy 26
16.2 Data Management 26
16.3 Quality Assurance 28
16.4 Data and Safety Monitoring 28
17.0 REFERENCES 28
18.0 APPENDICES 37

1.1 PROTOCOL SUMMARY AND/OR SCHEMA

With treatment advances, many patients live with advanced cancer and undergo cancer treatment for many years, similar to those with other chronic illnesses. Their symptom burden of pain, fatigue, and insomnia are high, accompanied by decreased quality of life and increased health care utilization. Opioid therapy is often a routine component of their care with evidence of some improvement, but there are no studies of long-term use of other medications or non-pharmacological approaches in this population. Our previous research has found that many patients living with advanced cancer are interested in integrative medicine therapies, such as acupuncture and massage, to improve symptom control and quality of life. With the widespread opioid epidemic ongoing in the United States, the Centers for Disease Control and Prevention (CDC),[1] The Joint Commission,[2] the American Society of Clinical Oncology (ASCO),[3] and the National Comprehensive Cancer Network (NCCN)[4] all recommend the use of non-pharmacological interventions, including acupuncture and massage, for pain management in adult cancer patients in adjunct to conventional care. However, high quality evidence about the comparative effectiveness and long-term durability of these two therapies for pain is limited. To inform patient decision-making, we propose an **I**ntegrative **M**edicine for **P**ain in Patients with **A**dvanced **C**ancer **T**rial (**IMPACT**) to answer the following patient-centered questions:

- **Patient Question 1:** Which of the treatments (acupuncture or massage) is more effective for pain and co-morbid symptoms (i.e. fatigue, insomnia and quality of life) in individuals living with advanced cancer?
- **Patient Question 2**: Given my situation, which of the two treatments is better for treating pain in patients like me?

Table 1. Protocol Summary

Study Title	**I**ntegrative **M**edicine for **P**ain in Patients with **A**dvanced **C**ancer **T**rial (**IMPACT**)
Specific Aim 1	To compare the effectiveness of acupuncture versus massage for pain and co-morbid symptoms in patients living with advanced cancer.
Specific Aim 2	To identify patient-level demographic characteristics (e.g. sex, race, age), clinical factors (e.g. insomnia, pain severity), and psychological attributes (i.e. outcome expectation) that are associated with a greater reduction in pain for either acupuncture or massage.
Patient Population	Patients experiencing chronic musculoskeletal pain for one month or greater
Total Enrollment	300 patients
Study Design	Two-arm parallel (Acupuncture vs. Massage) randomized controlled trial
Treatment	Participants will receive up to 10 treatments in the first 10 weeks (+/- 4 days) and then receive monthly booster treatments (+/- 7 days) for up to 26 weeks.
Time to Completion	Participants will be on the study for 26 weeks.

Figure 1 IMPACT Study Schema

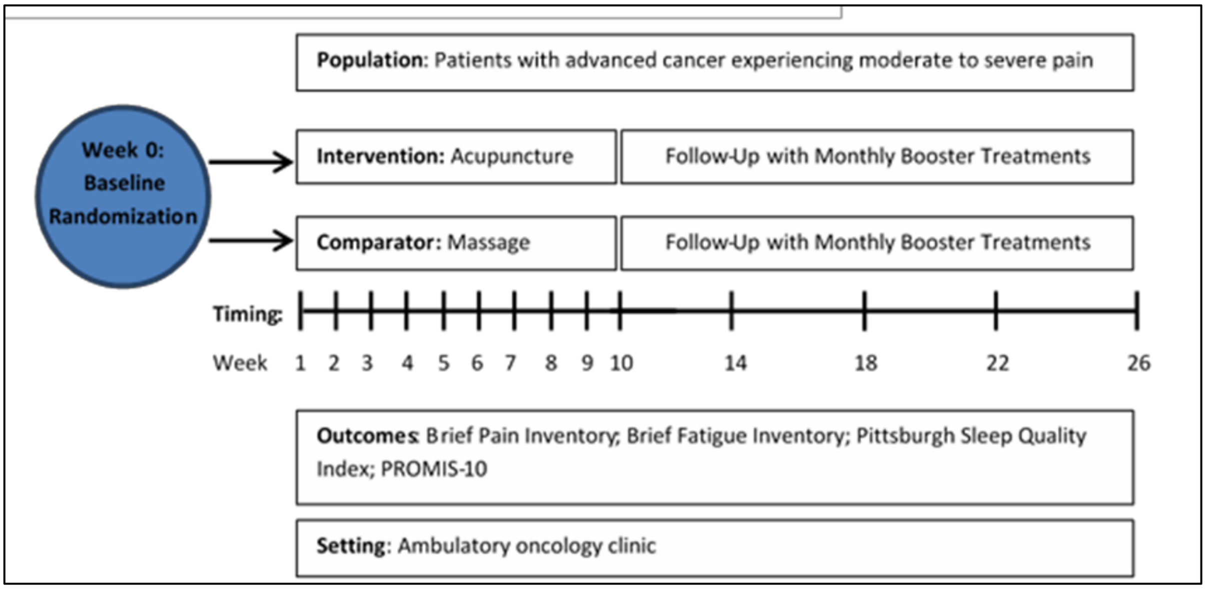

2.1 OBJECTIVES AND SCIENTIFIC AIMS

To inform patient decision-making, we propose an **I**ntegrative **M**edicine for **P**ain in Patients with **A**dvanced **C**ancer **T**rial (**IMPACT**) to answer the following study objectives:

- **Aim 1:** To compare the long-term effectiveness (26 weeks from randomization) of acupuncture versus massage for pain (primary outcome) and co-morbid symptoms (fatigue, sleep disturbance, and quality of life) in patients living with advanced cancer.
- **Aim 2:** To identify patient-level demographic characteristics (e.g. sex, race, age), clinical factors (e.g. insomnia, pain severity), and psychological attributes (i.e. outcome expectation) that are associated with a greater reduction in pain for either acupuncture or massage.

3.0 BACKGROUND AND RATIONALE

3.1 Burden of Cancer and Pain: Cancer is a leading cause of morbidity and mortality, second only to heart disease.[5] Early detection and advanced treatments such as hormonal therapies, targeted therapies, and immunotherapies have transformed cancer from a uniformly terminal illness into an illness that can be cured for some or chronic for many more. Recent estimates show that 15.5 million Americans are living with a cancer diagnosis, and this number is expected to exceed 20 million by 2020.[6] Compared with the general population, patients with advanced cancer are at a greater risk for chronic physical and psychological symptoms.[7-10] Pain is one of the most common symptoms among individuals with advanced cancer with prevalence rates as high as 66%.[11,12] Because of recent advances in cancer therapeutics, the definition for the extent of cancer is challenging because some metastatic cancer can now be "cured" or at least go into long-term remission leaving patients to often live with symptomatic sequelae. For the current protocol, we define advanced cancer as solid tumors that are un-resectable, locally invasive, or with metastases

that require ongoing oncological follow up and treatment. As consistent with the funding announcement, we will focus on the population of patients with advanced cancer that have treatable disease rather than those who require hospice care.

Pain has been shown to co-occur with fatigue and sleep disturbance and negatively influence health-related quality of life in patients with cancer.[4,13-16] For example, a study among 2,862 patients with cancer investigated the relationships between self-reported sleep difficulty, pain, and emotional distress and found that individuals reporting significant pain were 2.7 times more likely to experience sleep difficulty than those without pain.[17] We recently found that among 1,103 women with breast cancer receiving hormonal treatment, pain severity was significantly correlated with fatigue ($r=0.48$, $p<0.001$) and insomnia ($r=0.39$, $p<0.001$).[18] Among patients with advanced cancer, symptoms of pain, fatigue, and insomnia are the most commonly reported, often cluster together, and are generally not well managed.[9,10,19-23] Since pain is often associated with sleep disruption, fatigue, and diminished quality of life, we will carefully examine these co-morbid symptoms and key domains in quality of life as important secondary outcomes in the relationship to pain and to the proposed interventions.

3.2 Need for Non-Pharmacological Interventions: Historically, pain management in cancer has predominantly relied on drug therapies; however, increasing clinical evidence suggesting the potential harm over time of long-term opioid therapy for chronic cancer pain, not to mention the current widespread opioid abuse epidemic sweeping the United States, underscores a need for additional treatments.[3,24] Among patients with advanced cancer, high opioid use (≥5 mg oral morphine equivalents (OME)/day) has been shown to be associated with shorter overall survival, even after adjusting for age, sex, and prognostic group.[25] During our patient engagement, a wife of a patient living with stage IV prostate cancer told us, “We really would like something in addition to drugs to manage my husband’s pain so he is not drowsy all the time and can enjoy his time with family.” Another woman with advanced lung cancer who responded well to nivolumab (immunotherapy) said, “I don’t want to constantly feel that I am on a drug. I have always been sensitive to drugs. If there are things more natural, I will go for it.” As more individuals with advanced cancer live longer, patient-centered pain management integrating non-pharmacological interventions based on research evidence has strong potential to improve the quality of life for this population.

3.3 Acupuncture, a therapy of traditional Chinese medicine (TCM), involves penetrating the skin with thin, solid, metallic needles that are manipulated by hand or electrical stimulation.[26] Acupuncture is considered to be extremely safe with few side effects (e.g. needling pain, bruising).[27] Although not completely understood, the mechanism of acupuncture involves modulation of neurotransmitters including endogenous opioids in the brain, providing mechanistic plausibility as a treatment for pain.[28] With respect to the efficacy of acupuncture for chronic pain, in a patient-level meta-analyses of randomized controlled trials (RCTs), including approximately 18,000 to 21,000 patients with chronic non-malignant pain, acupuncture was found to be substantially better than usual care or standard care, significantly better than sham acupuncture, and approximately 90% of the effects of acupuncture relative to controls were sustainable at 12 months.[29-31] Although research is more limited in cancer populations, a systematic review and meta-analysis found that when acupuncture is incorporated into conventional cancer care, it is more effective than conventional drug management alone for cancer pain.[32] A recent well-done phase III randomized trial found that acupuncture was significantly more efficacious for pain reduction than sham control and usual care among breast cancer survivors (N=226), and the effect

was maintained up to 6 months.[33] Finally, there is some evidence suggesting that acupuncture may improve sleep disturbances, fatigue, and anxiety in cancer patients experiencing pain.[33,34]

3.4 Massage, which involves the manual manipulation of muscles and other soft tissue areas of the body, is one of the earliest known forms of pain relief. When practiced by trained professionals, massage is considered to be safe for cancer patients with no serious adverse side effects reported.[35] Since massage therapy techniques promote joint flexibility, relieve muscular tension, and improve range of motion, massage therapy has mechanistic plausibility for addressing musculoskeletal pain in patient populations.[24,36] In addition, massage creates a relaxing response, which may allow patients with pain to enhance their psychological coping.[37] In a recent meta-analysis conducted by the Evidence for Massage Therapy Working Group, massage therapy was effective at treating pain compared to other controls (such as reading, usual care, or active attention) (SMD=-0.55) in cancer populations.[35] These results are similar to the meta-analysis findings for patients experiencing pain in the general population showing that massage therapy was effective at treating pain compared to no treatment (SMD=-1.14).[38] However, the long-term persistence of massage's effects is somewhat unclear. A previously conducted RCT among individuals with low back pain (N=401) found that the effects of massage were durable for up to six months compared to usual care.[39] However, a recent RCT among patients with knee osteoarthritis (N=222) showed that massage was significantly more effective at eight weeks compared to either light touch or usual care. While the effects of massage persisted long-term, all three groups improved so the group difference was no longer significant at 52 weeks.[40] Lastly, in addition to pain management, massage therapy may improve fatigue, sleep, and anxiety in cancer populations.[35,37,41-43]

3.5 Gaps in Evidence: Based on the growing evidence of acupuncture[31,44,45] and massage[35,46] for the treatment of chronic pain, leading medical organizations such as the CDC,[1] The Joint Commission,[2] the American College of Physicians,[47] ASCO,[3] and NCCN[4,48] recommend non-pharmacological interventions in conjunction with drugs for pain management.[3,4,48] However, despite acupuncture and massage therapy both being widely-available and commonly-used non-pharmacological treatments for pain,[24,49] there is currently a ***gap in the evidence*** regarding the comparative effectiveness of these options for patients living with advanced cancer. As the ASCO guideline states, "There were no compelling data to recommend one of these therapies over another";[3] this presents significant uncertainty for patients and clinicians as they attempt to select the most effective treatment. Both acupuncture and massage often require a significant time commitment, travel, and cost; therefore, knowing the comparative effects between acupuncture and massage will readily inform patient and clinician decision-making.

Despite the growing evidence for the efficacy of acupuncture and massage for pain management, most trials in cancer populations have been short-term (<eight weeks) and with small sample sizes (N<100). Therefore, we know very little about the long-term durability of their treatment effects for pain in patients with cancer. Further, recent application of novel cancer therapy has transformed the lives of many individuals diagnosed with advanced cancer by allowing some to be cured and many more to live for years,[50] but often with a continued experience of ongoing pain and other co-morbid symptoms. However, previous non-pharmacological symptom intervention trials rarely include people with advanced cancer. In a recent NCI conference on acupuncture for symptom management in oncology, scientists

and stakeholders emphasized the need for "large and adequately powered trials with long-term follow-up to determine the definitive effects of acupuncture for common symptoms such as… **pain**, where there are promising signals from small trials."[51] Similarly, a recent white paper from the Pain Task Force of the Academic Consortium for Integrative Medicine and Health identified the need for research focused on the long-term therapeutic impact of evidenced-based non-pharmacological treatments for comprehensive pain care.[24] Further, despite the fact that advanced cancer patients with pain often experience fatigue and sleep disturbances,[52,53] little comparative effectiveness research has evaluated how different interventions affect these co-morbid symptoms in patients living with advanced cancer. We seek to conduct an RCT to evaluate the long-term comparative effectiveness of acupuncture versus massage for pain in patients living with advanced cancer. Our findings will provide patients living with advanced cancer and their health care providers with the needed evidence for patient-centered decision-making in choosing appropriate non-pharmacological treatment for pain.

3.6 Significance: Pain and co-morbid fatigue and sleep disturbance are among the most common and distressing symptoms for patients living with advanced cancer.[9,10,19-22] These co-occurring symptoms also negatively impact patients' quality of life and functional performance.[23,54,55] Unlike drug therapies that mostly focus on treating one symptom, acupuncture and massage can address multiple symptoms during treatment, which makes them potentially beneficial not only for pain but also for its related co-morbid symptoms (e.g. fatigue and sleep disturbance) among patients with advanced cancer. Despite tremendous patient interest, comparative effectiveness research in patients living with advanced cancer is very limited. This population has high symptom burden and represents a rapidly growing population thanks to novel treatment approaches such as targeted treatments and immunotherapies.

Acupuncture and massage are both widely available and commonly used non-pharmacological treatments for pain and other co-morbid symptoms in cancer populations. Among the 45 NCI-designated cancer centers, 89% recommend acupuncture and 84% recommend massage therapy for symptom management, which is 30% higher than six years ago.[49] Additionally, we previously found that cancer patients are more likely to use acupuncture and massage than those without cancer and the reasons for such use were for pain management.[56] Therefore, our proposed research will provide high quality evidence of the comparative effectiveness and durability of acupuncture versus massage that can be readily incorporated into clinical care to improve patient-centered decision-making. Thus, the findings of this study will have an immediate and substantial impact on millions of patients living with advanced cancer.

3.7 Preliminary Studies: The PI and investigative team have extensive content and methodological expertise as well as relevant experience performing patient-centered clinical research in the areas of symptom science and integrative oncology. This study is a logical extension of our previous work over recent years, which reflects the perspectives and participation of thousands of cancer patients.

3.7a Integrative Medicine and Pain Experience in Patients with Advanced Cancer: Our group has conducted a number of mixed-methods research studies to understand[57,58] and quantify[59-61] the expectation and unmet needs of patients and their preferences for integrative approaches in the context of conventional cancer care. In our recent survey study of almost

1,000 cancer patients (45% with advanced cancer), a majority of them expected integrative therapies to reduce pain and help them cope with the experience of living with cancer.[59] In another cross-sectional survey study among over 600 cancer patients seen in both academic and community hospitals (46% with advanced cancer), we found that 68% of them reported moderate to severe pain, and 78% reported moderate to severe fatigue in the past seven days. Further, we found that pain and fatigue were significantly associated with reduced physical activity since cancer diagnosis.[62] In preparation for this study, we performed analyses restricted to the 284 patients with stage IV cancer: mean age 60 years (range 29 to 86, SD 10), 55% women, 81% White, cancer type (Breast 23%, Thoracic 21%, Gastro-intestinal 19.4%, Head/Neck 14.4%). The mean worst pain in the last seven days was 6.3 (SD 1.7), despite the fact that 48% of these patients were currently receiving opioids, 35% non-steroidal anti-inflammatory drugs, 32% acetaminophen, 16% neuroleptic, and 16% anti-depressant treatments. This level of pain indicates substantial symptom burden and unmet pain management need in this population. Further, among this population, 68% indicated their willingness to participate in an acupuncture clinical trial, if offered.

3.7b Acupuncture for Pain and Co-morbid Symptoms: We completed an RCT of electro-acupuncture (EA) compared to sham acupuncture (SA) and waitlist control (WLC) in 67 postmenopausal women with breast cancer who self-attributed their arthralgia to taking AIs.[63] Acupuncturists delivered ten treatments of either EA or SA over the course of eight weeks. The primary aim was to assess the patient's pain intensity as measured by the Brief Pain Inventory (BPI) between EA and WLC. Of the 67 patients randomized to the three arms, 21 (95.4%) in the EA group and 20 (90.5%) in the SA group received all ten treatments. Only eight (12%) were lost to follow up by Week 12. Only a few minor adverse events, such as needling pain and bruises, were reported. At Week 8, the EA group had a clinically and statistically significant reduction in pain intensity (-2.2 vs. -0.2, Cohen's *d*=0.76, p=0.0004) and pain-related interference (-2.0 vs. 0.2, Cohen's *d*=1.04, p=0.0006) compared with the WLC. By Week 12 (four weeks after the end of treatment) the pain intensity scores continued to improve for the EA group and got worse for the SA group.[63] In addition, EA produced a consistent, clinically important pain reduction (greater than 30%) for all participants, while SA was only effective for participants who entered the trial with high expectations.[64] Further, compared to WLC, EA produced significant improvements in fatigue (p=0.0095), anxiety (p=0.044), and depression (p=0.015), and non-significant but marginal improvement in sleep disturbance (p=0.058) during the 12-week intervention and follow-up period. The Cohen's *d* values for pain and secondary outcomes suggest a moderate to large effect size for these outcomes, which demonstrates clinical utility. These data not only demonstrate the potential of EA for reducing pain and co-morbid symptoms in patients with cancer, but also demonstrate our ability to successfully implement and complete an acupuncture trial with few drop-outs.

3.7c Oncology Massage for Pain and Co-morbid Symptoms: While massage is one of the most popular integrative medicine approaches for the general population and patients with cancer,[56] its appropriate integration in hospital settings remains to be formally evaluated. We recently developed, implemented, and evaluated an integrative oncology massage program at the University of Pennsylvania for breast cancer patients receiving chemotherapy treatment in chemo-infusion suites.[37] Licensed massage therapists with advanced training in oncology massage administered massage sessions that lasted an average of 20 minutes (range, 15-30 minutes). Massage therapists delivered either head/neck or lower extremity massages and used light or very light compressions. Of 1,090 massage sessions offered,

692 (63%) were accepted. Patients were asked pre- and post-massage to complete an adapted Distress Thermometer scale (0-none to 10-extreme) for pain, fatigue, and anxiety symptoms. Patients self-reported outcomes demonstrated significant decreases in all three symptoms post-massage: pain (3.3 to 1.9), fatigue (4.8 to 3.0), and anxiety (3.9 to 1.7) (all Ps<0.001).[37] Additionally, 93% of patients stated they were satisfied/very satisfied with the massage, and 94% would recommend the massage program to another patient receiving chemotherapy treatment. Using qualitative feedback from patients who received a massage, the major themes describing their massage experience included relaxation, symptom relief, distraction, and a positive relationship with the massage therapist.[37] No negative adverse effects were reported. After the study, the health system incorporated this model to support symptom control for both breast and gynecological cancer patients.

4.1 OVERVIEW OF STUDY DESIGN/INTERVENTION

4.2 Design

The **I**ntegrative **M**edicine for **P**ain in Patients with **A**dvanced **C**ancer **T**rial (**IMPACT**) is a two-arm, parallel group, randomized controlled trial (RCT) to compare the effectiveness of acupuncture and massage for pain and co-morbid symptoms in a heterogeneous sample of 300 patients living with advanced cancer who have been experiencing moderate to severe pain (defined as self-reported worst pain in the past week as 4 or greater on a 0-10 numerical rating scale, based on the Brief Pain Inventory "worst pain" item). Eligible patients will be randomly assigned to acupuncture or massage using computer-generated numbers stratified on any current opioid use (Yes/No) and MSK site (Manhattan/Regional). Patients will receive weekly acupuncture or massage treatments for 10 weeks followed by monthly booster sessions up to 26 weeks. All patients will continue to receive their standard medical care and pain management as prescribed by their physicians. Patients will complete validated patient-reported outcome (PRO) measures of pain and co-morbid symptoms at seven time points: weeks 0 (baseline), 4, 10, 14, 18, 22, and 26.

4.3 Intervention

Patients will receive up to ten treatments during the first ten weeks and then receive monthly booster treatments of a similar type and duration for up to 26 weeks. The rationale for such treatment frequency is supported by both our prior work[63] and that of others.[65] We will allow 60 minutes for the initial treatment (including history taking, building rapport, and therapy) and 30 minutes for the follow up treatments to ensure equal contact time in both treatment groups. Further, a recent study suggests that a 30-minute massage produces similar pain reduction as a 60-minute massage in an oncology setting, further justifying our proposed treatment duration.[42] Additionally, before each massage or acupuncture treatment, the clinician will review the patient's medical chart to check the most recent platelet count value. As in our current clinical practice, if the patient has a platelet count below 15,000, the clinician will modify his/her techniques. In the case of acupuncture, shallow needling with minimal stimulation will be used, and needles will only be placed in the extremities. For patients with electronically charged medical devices, no stimulation will be used. In the case of massage, light touch will be used, and areas of bruising will be avoided. The clinician will document any treatment modifications and the medical reason for the modification in the patient's chart, which will allow us to systematically capture patients who received a modified

treatment. The clinician will also carefully document any potential adverse events such as bruising or bleeding. (See Appendices 1 and 2 for details of the treatment interventions).

4.2a Acupuncture Procedure: The treatment protocol has been developed by the PI over the last decade in collaboration with American and Chinese acupuncturists and has demonstrated improvements in pain, fatigue, and sleep among patients with cancer.[34,63] During acupuncture treatment, patients will lie comfortably on a table to receive acupuncture. The acupuncturist will prep the skin with 75% alcohol, wiping each point prior to needle insertion. The acupuncturist will place between 10 and 20 needles (30 mm or 40 mm and 0.16mm to 0.25 mm gauge, Seirin-America Inc., Weymouth, MA) at a minimum of four local points around the body area with the most pain and at individual points depending on the patient's co-morbid symptoms. The acupuncture needles will be inserted to appropriate depths depending on the location on the body and body type of the patient.[66] The acupuncturist will manipulate the needles to achieve the "De Qi" sensation for the patients. "De Qi" is a local sensation of soreness, numbness, or distension that accompanies the insertion and manipulation of needles during acupuncture.[67] The needles at the four local points for pain will be electrically stimulated at 2 Hz by connecting to a TENS unit. Electro-stimulation of needles is a common procedure in acupuncture clinical practice.[66,68] Our decision to use electro-stimulation of needles is based on physiological findings that low frequency electro-stimulation of acupuncture points stimulate the brain to release beta-endorphins.[69,70] Additionally, this type of electric stimulation has been shown to produce substantial effects in trials of acupuncture for osteoarthritis.[71,72] If the patient has an electronically charged device, they will not receive TENS stimulation. The acupuncturist will leave the needles in place for 20 minutes with brief manipulation at the beginning and end of the treatment. In our pilot studies, this manualized protocol was found to be well tolerated with a clinically important change in pain (greater than two-point reduction in pain-intensity).[63,73] As in our oncology practice, acupuncturists will avoid needling areas around port placement, tumors, or bone metastasis.

4.2b Massage Procedure: The PI developed the massage treatment protocol in collaboration with oncology clinicians and highly experienced oncology massage therapists. Our protocol demonstrated improvements in pain and fatigue among patients with cancer undergoing chemotherapy.[37] During massage treatments, patients will either lie comfortably on a table or sit comfortably in a chair to receive a massage focused on their primary area of pain. Consistent with oncology massage practice, therapists will administer compressions with light to moderate pressure and will use any of the following oncology massage techniques: compression; muscle stripping; active/passive range of motion, post-isometric stretching; effleurage (gliding); myofascial release; positional release; and trigger/tender point release.[74,75] Therapists will start with a five-minute protocol including guided diaphragmatic breathing exercise, rib mobilizations and OA release to increase parasympathetic tone. Next, depending on the patient's primary area of pain, the therapist will focus 20-minutes of massage on that specific body area followed by effleurage toward the heart. The massage therapist will focus on the following identified areas of pain: head/jaw; cervical spine; thoracic spine; shoulder; upper extremity; lumbar; sacral; pelvic; hip; and lower extremity. Any remaining time can be spent with integrative work to address global patterns noted in postural and gait assessment. As in our current oncology practice, therapists will avoid tissue manipulation areas around port placement, tumors, or bone metastasis.

4.2c Fidelity of Delivery of Interventions: Licensed and oncology-experienced

acupuncturists and massage therapists will deliver all treatments. All acupuncturists and massage therapists will be trained by the PI about the specific research protocol and educated on the importance of adherence to protocol methods and documentation of treatment visits. Our lead acupuncturist and massage therapists will observe and evaluate the study therapists twice a year. The lead therapists will also review at least two charts for each therapist per week for adherence to treatment protocol and documentation standards. They will communicate with the PI weekly regarding the quality monitoring of the treatments. If a new acupuncturist/ massage therapist joins the study protocol, they will be trained by their respective lead therapist. We have extensive experience in conducting integrative medicine symptom trials including ensuring the quality of interventions.[34,37,63,76]

5.0 THERAPEUTIC/DIAGNOSTIC AGENTS & NON-THERAPEUTIC ASSESSMENTS

Acupuncture Needles: 30mm or 40mm and 0.16mm - 0.25 mm gauge Seirin acupuncture needles will be used in this study. The needles are purchased and distributed from Seirin® in the United States (http://www.seirinamerica.com). Seirin acupuncture needles are approved by the FDA (http://www.accessdata.fda.gov/cdrh_docs/pdf/K962809.pdf).

6.1 CRITERIA FOR PARTICIPANT ELIGIBILITY

We have established broad eligibility criteria to be consistent with a pragmatic design, while ensuring the safety of participants and the rigor of clinical research. In brief to be eligible for the study, patients will be 18 years or older; have an advanced cancer diagnosis; have musculoskeletal pain for at least one month; and have moderate to severe pain in the past week. The below inclusion and exclusion criteria are based on our own research[63] and existing literature[77,78] and informed by pain and oncology physicians and researchers who are familiar with pain management in ambulatory oncology settings. Prior pain and symptom control research in patients living with advanced cancer included both patients with solid tumors that spread beyond lymph nodes or hematological cancers. From our clinical and research experience, we believe that cancer type does not affect the treatment response to acupuncture or massage for patients with musculoskeletal pain. We selected these criteria to ensure that: 1) the study is safe for the research participants; 2) the population is relevant to the eventual dissemination of the information with as wide as possible inclusion criteria; and 3) the population is relatively well-defined so that the change in outcomes among intervention groups can be appropriately measured and detected. No individuals will be excluded on the basis of race, ethnicity, or sex.

6.2 Participant Inclusion Criteria

- Age ≥ 18 years or older
- Having a diagnosis of the following: stage III or IV lung cancer; any stage pancreatic cancer; unresectable cholangiocarcinoma; unresectable liver cancer; unresectable ampullary or peri-ampullary cancer or other stage IV gastrointestinal cancer; stage III or IV ovarian or fallopian tube cancers or other stage IV gynecologic cancer; stage IV breast cancer; stage III testicular cancer; stage IV genitourinary cancer; stage III or IV sarcoma; stage IV melanoma; stage III or IV head/neck cancer; stage IV endocrine cancer; or hematological malignancies (lymphoma, myeloma, and leukemia)

- Be ambulatory (Karnofsky functional score of ≥ 60)
- Having musculoskeletal pain, defined as regional (joints, extremities, back, neck) or more generalized (fibromyalgia or chronic widespread pain); Patients with a neuropathic component to their pain that involves the extremities or back will be eligible.

- Having musculoskeletal pain for at least 1 month
- Having had pain for at least 15 days in the preceding 30 days
- Having a pain rating of 4 or greater in worst pain on a 0-10 numerical rating scale in the preceding week
- Having an expected prognosis of greater than six months as judged by the treating oncologist or study physician

6.3 Participant Exclusion Criteria

- Having a platelet count <15,000
- Cognitive impairment precluding response to study assessments
- Unwilling to accept random assignment
- Unwilling to commit to the 26-week study time period
- Have non-musculoskeletal pain syndromes (headache, facial pain, chest pain, visceral abdominal pain) if these are the sole source of pain but can be present as co-morbid conditions as long as a patient has a primary musculoskeletal pain condition defined as above.

7.0 RECRUITMENT PLAN

Recruitment Plan (with Limited waiver of Authorization)

Our primary recruitment approach will be via sending recruitment letters to potential participants. Potential patients who meet basic eligibility criteria will be identified via querying of Dataline at MSK and sent a recruitment letter (See Appendix 3). The recruitment letter introduces the study to potential participants and states that we are conducting a study to compare the effectiveness of acupuncture versus massage for individuals diagnosed with musculoskeletal pain and if interested in learning more about the study, the patient should contact the research team. The letter provides patients with an opt-out phone number and study e-mail address to contact if they do not wish to participate or be contacted further. We will also be identifying patients that meet basic eligibility criteria and have reports of pain on the MSK Engage symptom questionnaire through a Dataline query. We have successfully used this recruitment method for similar studies (IRB Protocol #16-1579).

In addition to sending recruitment letters, potential participants also can be identified and referred to the study clinical research coordinator (CRC) for accrual and consent by protocol investigators. The study PI and other members of the research team will reach out to colleagues about the study and present at Service meetings, including Breast Medicine, GI Oncology, GU Oncology, Head and Neck Oncology, GYN Med Oncology, Nursing, and Psychiatry and Behavioral Sciences to introduce the study. Also, colleagues in Survivorship will be informed about the study, and recruitment materials will be provided to them. In addition to Integrative Medicine physicians, other Integrative Medicine therapists can also refer patients to the study. Study investigators and interested colleagues will be provided with study flyers and/or rack cards to provide to potential participants (See Appendices 4 and 5 for a study flyer and rack card). Potential participants may also be self-referred or referred by a clinician from other hospitals. Information about the protocol will appear in lay language on MSK's website and on clinicaltrials.gov. Printed materials will be posted in clinic areas where we have successfully posted study materials for other Integrative Medicine studies before

(e.g., the Breast and Imaging Center, the Main Hospital, Kimmel, and the Rockefeller Outpatient Pavilion). Permission from the clinic sites will be obtained before posting in any location. Materials will also be distributed to potential referral sources who we have worked with on other research studies. All study recruitment materials will be submitted to, and approved, by the Institutional Review Board. We have used these recruitment strategies successfully to recruit participants to similar studies (IRB Protocol #16-1579).

Initial contact with potential participants typically will be made by a member of the study team. The recruitment process presents no more than minimal risk to patient privacy, and minimal PHI will be maintained on screening logs. For these reasons, we seek a (partial) limited waiver of authorization to: (1) review MSK patient medical records to identify potential research subjects and obtain information relevant to the enrollment process; (2) converse with patients regarding possible enrollment; (3) handle PHI contained in those records and provided by potential subjects; and (4) maintain minimal PHI information in a screening log of patients approached.

Once a patient is deemed eligible, they may be enrolled through an in-person consent appointment, econsent or by a verbal consent conducted over the phone. Patients who do not have a desktop or laptop computer capable of a remote econsent and who cannot travel to Manhattan may be consented using a verbal consent. MSK participants, solely, will have the option of a verbal consent. Baptist Alliance MCI participants will not have the option of a verbal consent and will only have the written consent option.

To encourage completion of all study procedures, participants will receive $40 for completing the Week 10 assessment and $60 for completing the Week 26 assessment (total of $100). Individuals who withdraw from the study will be compensated for the assessments they have completed.

7.1 Research Participant Registration

Confirm eligibility as defined in the section entitled Inclusion/Exclusion Criteria. Obtain informed consent, by following procedures defined in section entitled Informed Consent Procedures. During the registration process registering individuals will be required to complete a protocol specific Eligibility Checklist. The individual signing the Eligibility Checklist is confirming whether or not the participant is eligible to enroll in the study. Study staff are responsible for ensuring that all institutional requirements necessary to enroll a participant to the study have been completed. See related Clinical Research Policy and Procedure #401 (Protocol Participant Registration).

7.2 Randomization

Participants will be randomized to acupuncture or massage using MSK's Clinical Research Database (CRDB), a secure computer system that ensures full allocation concealment. After eligibility is established and consent is obtained, patients will be registered through the Clinical Trials Management System (CTMS) and then randomized using the Randomization Module in the CRDB. Randomization will be accomplished by the method of random permuted block stratified by any current opioid use (Yes/No - any amount used in the last week) and by each MSK site (Manhattan, Basking Ridge, Bergen, Monmouth, Nassau,

Suffolk-Commack, Westchester, and Miami Cancer Institute).

Information on group assignments will be communicated to the CRCs who schedule treatment and follow up visits, acupuncturists, and massage therapists. The study statisticians and the outcome assessment CRC will remain blinded. We will accomplish the randomization in two steps. First, the CRC will inform the subject whether s/he is randomized to the acupuncture or massage group after the baseline visit. Second, the CRC will inform the treating acupuncturist/ massage therapist about subject randomization assignment.

8.1 INFORMED CONSENT PROCEDURES

Before protocol-specified procedures are carried out, consenting professionals will explain full details of the protocol and study procedures as well as the risks involved to participants prior to their inclusion in the study. Participants will also be informed that they are free to withdraw from the study at any time. All participants must sign or verbally agree to an IRB/PB-approved consent form indicating their consent to participate. This consent form meets the requirements of the Code of Federal Regulations and the Institutional Review Board/Privacy Board of this Center. The consent form will include the following:

1. The nature and objectives, potential risks and benefits of the intended study.
2. The length of study and the likely follow-up required.
3. Alternatives to the proposed study. (This will include available standard and investigational therapies. In addition, patients will be offered an option of supportive care for therapeutic studies.)
4. The name of the investigator(s) responsible for the protocol.
5. The right of the participant to accept or refuse study interventions/interactions and to withdraw from participation at any time.

Before any protocol-specific procedures can be carried out, the consenting professional will fully explain the aspects of patient privacy concerning research specific information. In addition to signing the IRB Informed Consent, all patients must agree to the Research Authorization component of the informed consent form. If a verbal consent is being conducted, the consenting professional will use the IRB/PB-approved verbal informed consent script when calling patients. If the patient agrees to participate, the consenting professional will sign and date the verbal consent and a copy will be sent to the patient. A verbal consent would be used in cases where individuals do not have access to a c omputer and those who are unable to travel to Manhattan for an in-person consent appointment. This research involves no more than minimal risk to participants and use of a verbal consent would not adversely affect the rights and welfare of the research participants.

Each participant and consenting professional will sign or verbally agree to the consent form. The participant must receive a copy of the signed or verbal informed consent form, and a copy of the signed or verbal consent form will be sent to the patient's EMR. Additionally, a member of the research staff will notify the primary oncologist of the patient's enrollment in the study.

9.0 PRE-TREATMENT/INTERVENTION

9.1 Initial Screening: All potential participants will undergo an initial screening with a CRC in person or over the telephone. At this initial contact, the CRC will explain the study goals and procedures and ensure that participants meet basic eligibility criteria.

9.2 Clinician Screening, Informed Consent, and Baseline Assessment: Interested and potentially eligible patients will be scheduled to meet with physicians or nurses for a screening visit to confirm eligibility, including a diagnostic history/physical for musculoskeletal pain and a self-reported questionnaire about the patient's pain experience. This screening form may be completed online using REDCap (Research Electronic Data Capture) or over the phone by a member of the research staff within two weeks of enrollment. If deemed eligible, the clinicians and/or study staff will explain the study procedures and review the written informed consent with the patient. After patients sign the informed consent, they will complete a set of baseline questionnaires. Please see Table 2 below for questionnaires collected throughout the study period. All questionnaires and diaries have a window of plus or minus ten days.

Table 2. Schedule of data collection

Outcome	Active Intervention			Follow Up			
	Week 0	Week 4	Week 10	Week 14	Week 18	Week 22	Week 26
**Primary Outcome**							
Brief Pain Inventory*	X	X	X	X	X	X	X
**Secondary Outcomes - Fatigue, Sleep, and Quality of Life**							
Brief Fatigue Inventory	X		X		X		X
Insomnia Severity Index	X		X		X		X
Hospital Anxiety and Depression Scale	X		X		X		X
PROMIS-10 Global Health	X		X		X		X
Patients' Global Impression of Change			X		X		X
**Covariates**							
Pain History	X						
Demographics (e.g., age, sex, race/ethnicity)	X						
Clinical Characteristics (e.g., tumor type, stage, cancer therapy)	X		X		X		X
Pain Medication Diary	X	X	X	X			X
**Predictive Variables**							
Mao Expectancy of Treatment Effects	X		X				

* Worst pain severity item is the primary outcome

9.3 Covariates: We will collect specific demographic (e.g. age, sex, race/ethnicity) and other relevant historical medical data on each subject (e.g. cancer treatment). We will also track their use of analgesic medications (e.g. acetaminophen, non-steroidal anti-inflammatory drugs, opioids, and adjuvants for neuropathic pain) by having patients complete weekly pain

medication diaries at Weeks 0, 4, 10, 14 and 26 to calculate weekly average analgesic medication usage throughout the study time period.[79] IRB deviations will only be reported for these pain diaries if they are not returned or are returned with 5 or more days missing. As pain often results in increased health care utilization, we will track emergency department visits and hospitalizations via EHR. Additionally, we will collect the patient's reasons for either stopping treatment or dropping out of the clinical trial, such as treatment adverse events, disease complications, or scheduling issues with work.

10.0 TREATMENT/INTERVENTION PLAN

Subjects will receive acupuncture and massage treatments at MSK's Bendheim Integrative Medicine Center (1429 First Avenue at 74th Street) and/or at the Breast and Imaging Center (300 East 66th Street at 2nd Avenue) and/or Brooklyn Infusion Center (557 Atlantic Ave). The OneMSK sites of MSK Westchester (500 Westchester Avenue), MSK Commack (650 Commack Road), MSK Basking Ridge (136 Mountain View Blvd), MSK Monmouth (480 Red Hill Road), MSK Nassau (1101 Hempstead Turnpike) and MSK Bergen (225 Summit Avenue) will be options for patients to receive these treatments once they are approved internally. Each participant will receive up to ten treatments of either acupuncture or massage during the first ten weeks and then receive monthly booster treatments of a similar type and duration for up to 26 weeks. All Integrative Medicine Service acupuncturists and massage therapists are licensed, credentialed employees of MSK.

11.0 EVALUATION DURING TREATMENT/INTERVENTION

The study schema and study schedule were presented in Section 1.0, Figure 1, and Section 9.0, Table 2, respectively. The following questionnaires will be collected according to the study schedule table (See Appendix 6). All patient-reported outcomes (PROs) have been previously validated and shown to be reliable, valid, and responsive to change in our prior studies.[34,80] The average time to complete the PROs is 30 minutes, which has been judged to be acceptable by our prior study participants with minimal missing data. Patients will complete PROs online using Research Electronic Data Capture (REDCap) or over the phone with the outcome assessment CRC, who is blinded to treatment group. To minimize missing data, the CRC will check surveys after completion. For patients who miss a study assessment, the CRC will email/call patients to complete the assessment. Additionally, for patients who are unable to complete all PROs (due to sickness, time constraints, etc.), we will ask them to complete only the Brief Pain Inventory questions (primary outcome). Using this approach, over 300 patients who are in our current research studies have completed PROs online using REDCap with less than 10% missing data. Online data collection through REDCap has been well received by our patient population. Also, our prior acupuncture study had only 4% loss to follow up at the active intervention and a total of 12.5% loss to follow up by Week 24.[81] The data collection schema can be seen in Section 9.0, Table 2.

11.1 Primary Outcome: Worst Pain Item from the short-form Brief Pain Inventory (BPI): The short-form BPI will be used to quantify pain severity and pain interference. The BPI contains 4 pain severity items and 7 pain interference items, all rated on a scale from 0 to 10 (higher ratings indicate worse pain intensity/interference). A pain interference subscale can be computed by taking the average rating of the 7 pain interference items. A pain severity

subscale score can similarly be computed for the 4 pain severity items; however, the Worst Pain severity item and the Average Pain severity item are often examined separately from the pain intensity subscale in clinical research because they tend to be more sensitive indicators of changes in patients' perceived pain. As such, the **primary outcome** of this study will be the patient's rating of their **Worst Pain** in the past week with response choices of 0 "no pain" to 10 "pain as bad as you can imagine." The Average Pain rating in the past week and the pain interference subscale will be used as secondary pain outcomes. The psychometrics of the BPI are well-established with Cronbach's alpha ranging from 0.77 to 0.91. The BPI is one of the most widely used instruments to measure pain in patients and has been demonstrated to be a reliable, valid, and responsive measure.[82] Farrar (Co-I) et al. found that a 30% or greater reduction in the pre-post intervention pain score is a clinically important change;[83-85] therefore, to enhance the interpretation of the pain outcome data, we will summarize response to acupuncture or massage using this criteria.

11.2 Other PROs:

Patients' Global Impression of Change (PGIC) is a one item survey that will be used to define a clinically important change in pain from the patient's perspective.[84,86] Patients will be asked "How would you describe your pain since the first clinical visit? I am: very much worse, much worse, a little worse, the same, a little improved, much improved, very much improved." Subjects reporting "much improved" and "very much improved" will be considered responders. The PGIC can be used as an anchor to derive anchor-based minimally important differences (MIDs) for pain measures like the BPI.

Brief Fatigue Inventory (BFI) will be used to determine the effect of treatments on fatigue. This 9-item instrument was designed to assess one construct of fatigue severity in cancer and non-cancer populations. Three items ask patients to rate the severity of their fatigue at its "worst," "usual," and "now" during normal waking hours, with 0 being "no fatigue" and 10 being "fatigue as bad as you can imagine." Six items assess the amount that fatigue has interfered with different aspects of the patient's life during the past 24 hours. The interference items are measured on a 0–10 scale, with 0 being "does not interfere" and 10 being "completely interferes." [87] A composite fatigue severity score can be found by averaging the 9 item scores. The score of the scale was found to be reliable and valid in multiple languages and diverse populations.[87,88]

Insomnia Severity Index (ISI) will be used to measure subjective insomnia severity.[89] The ISI has 7 items rated on a 5-point Likert response scale (e.g., 0 = no problem; 4 = very severe problem), yielding a total score ranging from 0 to 28 with higher scores representing more severe insomnia symptoms. The usual recall period is the "last month". The ISI authors suggest the following guidelines for interpreting the ISI total score: < 8, no clinically significant insomnia; 8-14, subthreshold insomnia; 15-21, clinical insomnia (moderate severity); > 21, clinical insomnia (severe). [89] The ISI has demonstrated internal consistency, reliability, construct validity, specificity and sensitivity in a representative sample of 1670 cancer patients.[90] The ISI has established minimally important change values to ensure that the change is not only statistically, but also clinically, meaningful to patients.[91] A reduction of eight points has been deemed to be clinically significant improvement.[91]

Hospital Anxiety and Depression Scale (HADS) will be used to explore the effect of treatments on psychological distress. HADS is a 14-item scale with 7 items measuring depression and 7 items measuring anxiety. Each item is answered by the patient on a four-

point (0-3) response category so possible scores range from 0-21 for anxiety and depression, with higher scores indicating higher symptomatology. Established cutoffs are: 0–7 not significant; 8–10 subclinical; and 11-21 clinically significant depression/anxiety.[92] Factor analysis showed two distinct but correlated factors of anxiety and depression.[93] The scale scores have been shown to be both reliable and valid.[94]

Patient Reported Outcomes Measurement Information System (PROMIS®) Scale v1.2 - Global Health is a brief instrument composed of 10 items that demonstrates adequate reliability and validity[95,96] as a measure of health related QOL in general and clinical populations.[97,98] Patients are asked to respond to questions 1-8 and 10 on a scale of 1-5. Question 9 is on a 0-10 scale (average pain rating). The measure yields two scores, Physical Health and Mental Health, that will be used as secondary outcomes to evaluate the effect of acupuncture on QOL.[95] These scores will be calculated using item-level calibrations based on item respone theory (IRT) scaling and then transformed to T-Scores, which are standardized such that 50 represents the mean for the US general population, and the standard deviation around that mean is 10 points. Higher scores indicate better Physical and Mental Health.

Mao Expectancy of Treatment Effects (METE) is a four-item instrument originally developed as the Acupuncture Expectancy Scale (AES) by Mao (PI) et al.[99] Outcome expectancy has long been considered an important predictor of treatment outcomes and has gained increasing recognition in clinical trials.[100,101] It has demonstrated reliability (Cronbach's α of 0.82) and validity and is positively correlated with patient self-reported efficacy and satisfaction.[99] The score ranges from 4 to 20, with higher scores indicating greater expectancy. We will use this measure to explore whether expectancy predicts treatment outcomes and may impact the observed differences between groups.

12.1 CRITERIA FOR REMOVAL FROM STUDY

Any subjects experiencing a serious adverse event (SAE) felt to be related to the study intervention will be removed from receiving further treatment. Patients also will be removed from receiving further treatment if they miss two consecutive treatment visits without notification of study staff, or if discontinuation from the treatment is deemed by the principal investigator to be in their best interest. Subjects discontinued from the treatment aspects of the clinical trial will be scheduled to continue the follow-up study assessments up to 26 weeks. Any subject withdrawing their consent to participate in the study or their authorization to use their protected health information will be withdrawn from the study.

Subjects will be informed during the consent discussion that treatment may be discontinued due to:

1) Intolerable side effects (side effects felt by the patient, acupuncturist, massage therapist, or physician to be of greater severity than the potential benefit from treatment);
2) Failure to attend 2 consecutive treatment visits without notification of study staff.

If patients fail to attend sessions with notification, every effort will be made to reschedule the patient such that they can receive the maximum number of treatments.

Reasons for subject discontinuation from the clinical trial will be documented on the Study Termination Form, along with any referrals that are made. We will make every effort to continue to collect data on every subject for the entire study duration regardless of whether or not the subject continues to adhere to the study interventions, assuming the subject has not withdrawn his/her authorization to obtain such information.

13.0 CRITERIA FOR OUTCOME ASSESSMENT AND ENDPOINT EVALUABILITY

13.1 Criteria for Therapeutic Response/Outcome Assessment

Our primary hypothesis is that over 26 weeks from randomization, acupuncture will result in greater overall improvements in pain (primary outcome), co-morbid fatigue, sleep disturbance, and quality of life compared to massage. Our primary outcome measure is BPI Worst Pain, which will be assessed at baseline and 4, 10, 14, 18, 22 and 26 weeks post-randomization. Our secondary outcome measures are the BFI, ISI, HADS, and the PROMIS-10 Global Health, which will be assessed at baseline and 10, 18 and 26 weeks post-randomization. Additionally, for individual patients, prior research supports that a 30% reduction in a patient's pain score pre-post treatment is considered a clinical response.[83-85] We will summarize response at the end of treatment on BPI Worst Pain using this criterion.

13.2 Criteria for Study Endpoint Evaluability

All patients who are randomized will be considered evaluable for the study primary endpoint of change in BPI Worst Pain. No patient will be replaced after randomization. We will, at the minimum, have the baseline BPI Worst Pain value for all randomized patients, since patients rate this item as part of eligibility screening. All randomized patients will be included in the analyses using the intention-to-treat (ITT) principle (i.e. participants will be analyzed according to the treatment group to which they will be randomly allocated regardless of drop-out or treatment adherence status). We will also perform a per-protocol sensitivity analysis among treatment and assessment completers.

14.0 BIOSTATISTICS

This is a two-arm, parallel group RCT to compare the effectiveness of acupuncture and massage for pain and co-morbid symptoms in a heterogeneous sample of 300 patients living with advanced cancer who have been experiencing moderate to severe pain. Patients will be randomized to receive acupuncture or massage, stratified by current opioid use (yes/no) and each MSK site (Manhattan, Basking Ridge, Bergen, Monmouth, Nassau, Suffolk-Commack, Westchester, and Miami Cancer Institute). Patients will receive weekly acupuncture or massage treatments for 10 weeks followed by monthly booster sessions up to 26 weeks. Patients will complete validated patient-reported outcome (PRO) measures of pain and co-morbid symptoms at seven time points: weeks 0 (baseline), 4, 10, 14, 18, 22, and 26. We expect to accrue approximately 11-13 patients per month, and we anticipate the study will be open to enrollment for approximately 27 months. We expect the total study duration to be 3 years.

We describe the analysis for each aim below using the intention-to-treat (ITT) principle (i.e. participants will be analyzed according to the treatment group to which they will be randomly allocated regardless of drop-out or treatment adherence status). We will also perform a per-

protocol sensitivity analysis among treatment and assessment completers, but study conclusions will be based upon the ITT analysis results. For all specific aims, our main

analytic tool will be linear mixed-effects models (LMMs) because our primary outcome (worst pain severity) and secondary outcomes (average pain severity, pain interference, fatigue, insomnia, psychological distress, and QOL) are repeated continuous outcomes over time.[102] This statistical procedure takes into account within-subject correlations from repeated measurements in the same subjects and allows estimation of between-group differences without necessitating exclusion of participants with missing data. The general template of each LMM will model the outcome as a function of treatment arm and assessment time, controlling for the randomization stratification variables (baseline opioid use and MSK site), and including a subject-specific random intercept and slope. We will tailor this general LMM template to test the specific aim hypotheses by adding interaction terms (e.g. time-by-intervention) and additional covariates of interest to the model, and by reparametrizing the assessment time variable to focus on specific contrasts.

14.1 Specific Aim 1: We will plot the outcome measure trajectories by randomization arm over time and summarize each outcome measure at each assessment time by treatment arm using descriptive statistics. Tests of ITT differences between randomization arms with respect to changes in outcomes will be based on coefficients from specific time-by-arm interactions added to the general LMM template described above. Our primary effectiveness comparison (Aim 1) will focus on changes in BPI Worst Pain from baseline to 26 weeks between acupuncture vs. massage. Aim 1 secondary outcomes (e.g. fatigue, insomnia, QOL) will be analyzed using the same methods described above. To evaluate the impact of pain medication use on our Aim 1 findings, we will conduct sensitivity analyses by adding time-dependent variables to our LMMs indexing patient opioid and non-opioid analgesic use at each assessment time. To enhance patient-centered data interpretation and decision-making, we will also perform responder analyses by considering those who experienced 30% or greater reduction in worst pain as responders at end of treatment/Week 10.[83-85] We will compare the proportion of responders in acupuncture and massage at the end of the intervention period using descriptive cross-tabulations and logistic regression adjusting for the randomization strata.

14.2 Specific Aim 2, Heterogeneity of Treatment Effect (HTE): An essential part of patient-centered care is recognizing that not all patients will respond to treatments the same way. We will conduct exploratory, hypothesis-generating HTE analyses to identify patient-level factors associated with treatment response to either acupuncture or massage by incorporating relevant variables (e.g. sex, expectation, opioid use) and variable-by-intervention interaction terms in linear regression models predicting week 26 worst pain controlling for baseline worst pain and stratification factors. Each variable of interest will be assessed for HTE in a separate model. For these exploratory regression analyses, we will guard against inflated type I error due to multiple testing by adjusting the variable-by-intervention interaction p-values for the false discovery rate.[103,104] Our current focus on evaluating and reporting HTE will be based on the approach proposed by Kent et al.[105] However, we will also apply promising emerging Bayesian[106,107] and machine learning[108,109] methods, which can identify HTE and subgroups based on multiple variables simultaneously and are potentially more powerful than traditional univariate methods. Since we expanded our inclusion criteria to allow patients with hematological malignancies to enroll, we will also perform exploratory subgroup analysis to see if there is any difference in treatment effect (both primary and secondary outcomes) among patients with solid tumor cancer versus blood cancer. Because our trial will enroll patients with advanced cancer, interventions may need to be modified for patient safety issues such as for those with low platelets or bruising in the area where there

is pain. We will conduct exploratory analyses to examine if there are any differences in outcomes for those patients who received non-modified treatments versus those who had modified treatments. We will also conduct exploratory analyses to see whether individuals with low platelet counts experienced more adverse events compared to patients with normal platelet counts.

14.3 Sample Size and Power: Our sample size will provide sufficient statistical power to detect clinically relevant effect sizes for our primary pain outcome between acupuncture vs. massage (Aim 1). Given that patients living with advanced cancer may have unanticipated health issues (e.g. hospitalizations, death), we conservatively anticipate loss to follow up to be 20% by 26 weeks. For our sample size/power considerations, we calculated the smallest standardized effect size (aka, Cohen's d) we will be able to detect with .80 power, given our sample size of 300 and other assumptions (see below). To estimate this smallest detectable effect size, we used the methods of Lu, Luo, & Chen (2008),[110] which describes sample size calculations for a class of analyses called the "mixed model for repeated measures" (MMRM) in two-arm randomized clinical trials with participant attrition. Our LMM analyses fall under the MMRM class of analyses. Using the "power.mmrm" function from the R package "longpower", we applied the formulas in Lu et al. (2008) to derive the smallest detectable effect size for the coefficient of the time-by-arm interaction term in our LMM (see Section 14.1), given our study design and assumptions, which we transformed to represent the standardized mean difference (aka, Cohen's d) between the two arms at 26 weeks post-randomization. Although these formulas allow for multiple repeated measurement times, we conservatively used only the baseline and 26-week timepoints for these calculations. With 150 participants in each of the two active intervention arms, we will have power of 0.80 to detect an effect size of 0.35 (standardized mean difference, Cohen's d) at 26 weeks post-randomization between acupuncture vs. massage, assuming 20% loss to follow up,[110] correlation between baseline and 26-week worst pain of 0.50, and two-sided alpha of 0.05. Based on our own preliminary data in patients with stage IV cancer who experienced moderate to severe pain (N=284), the mean worst pain score was 6.3 with SD of 1.7. A difference of 1 on the worst pain score (considered a clinically meaningful difference in pain) based on SD of 1.7 equals an effect size (Cohen's d) of 0.59. In this study, we have 99% power to detect this clinically meaningful mean difference of 1 point (Cohen's d of 0.59) on the BPI-Worst Pain score. However, as noted above, we are adequately powered to detect an effect size as small as d=0.35. Thus, our trial is more than sufficiently powered to detect a clinically meaningful difference between acupuncture and massage at 26 weeks. During engagement sessions, patients told us that a difference of 1 in worst pain severity would be considered clinically important for them to choose one treatment over the other (acupuncture vs. massage). This is also consistent with the Methods, Measurement, and Pain Assessment in Clinical Trials (IMMPACT) group's recommendation for interpreting the clinical importance of treatment outcomes in chronic pain clinical trials.[111] In addition, this is used in a recent high-impact publication comparing opioid vs. nonopioid medication for chronic low back pain.[112] In summary, this sample size estimation is supported by patient engagement, expert consensus from literature, and our own preliminary data in patients with advanced cancer who experience moderate or severe pain.[32,35,63,111,112]

14.4 Missing Data: As the only certain way to avoid biases from missing data is to collect complete data,[113] we will minimize the occurrence of missing observations by using a well-piloted clinical trial design and protocol, well-trained research staff, and an acceptable participant burden.[114] Our prior trial had only 12.5% attrition by Week 24.[81] By using monthly data collection time points throughout the trial, we will be able to collect patient-reported outcomes and engage patients on a regular basis to help retain patients throughout the 26-week study. Additionally, for patients who have time constraints regarding completing the

outcome assessments, we will ask them to only complete the primary outcome measure, the Brief Pain Inventory (less than two minutes required in our experience). We will ask those who withdraw from the treatment interventions to continue to provide data and we will reimburse them for completing the evaluation. Further, we will allow patients to enroll in the clinical trial regardless of their socio-economic status by covering the costs associated with the clinical interventions (acupuncture or massage) using the patients' own insurance coverage or philanthropic funds. We will also have evening and weekend treatments available to allow patients who work to enroll in the IMPACT study and schedule treatments around their work schedules to prevent missing treatments or data collection. Lastly, for those who voluntarily withdraw from the study, we will record their reasons for withdrawing. Because missing data is inevitable in a prospective study like this (due to hospitalization and potential death), our second line of defense is to perform sensitivity analyses (e.g. assess impact on results of adjusting for patient disease progression or death) and apply data analysis strategies that are as robust as possible to data losses. We will first explore whether missingness is associated with observed variables (particularly randomization arm and the baseline outcome measures) by comparing patients with complete and incomplete data. Of note, the LMMs described above validly include patients with incomplete data under the missing at random assumption. However, our exploration of the data may deem the missing at random assumption to be inappropriate. In this case, multiple imputation and pattern mixture models are well-established methods we will use to help us deal with these issues.[115,116] We will perform sensitivity analyses to evaluate the robustness of our LMM results by refitting the models after imputing the missing Week 26 outcomes using multiple imputation.

15.1 TOXICITIES/RISKS/SIDE EFFECTS

Potential Risks: Patients will be monitored for side effects at each visit. Adverse effects related to the administration of either acupuncture or massage will be collected each week before and after each treatment by the acupuncturist/massage therapist or CRC. CTCAE Version 5 will be utilized for toxicity evaluation.

The proposed research study is considered to be low risk. All potential risks that might occur as a result of participation will be detailed in an informed consent form and will also be fully discussed with each patient prior to enrollment. We will also explain to each patient that while some risks are not predictable, every precaution consistent with the best medical practice to protect the health and safety of subjects will be taken. We will document all adverse events and report any related serious adverse events promptly to the IRB.

Physical Risks of Acupuncture: Acupuncture has an established safety record. The most common side effects are mild pain on needle insertion, occurring at rates twice that of the placebo group. There is a possibility of a small amount of bleeding or bruising around the acupuncture sites. Other side effects include allergic reactions, drowsiness, anxiety or nervousness, vasovagal reaction symptoms (dizziness, fainting, nausea or vomiting) and, very rarely, skin infections at the site of insertion. On rare occasions, chest needling can lead to a pneumothorax, although this is extremely rare.

Physical Risks of Massage: Massage has an established safety record. The most commonly reported side effects are localized temporary fatigue and soreness. There is a risk of hematoma, embolism, bone and nerve injuries with massage, although it is extremely rare.

Psychological Risks: It is possible that subjects may be upset to find out that they are randomized to their non-preferred arm of the study. With appropriate consent and the debriefing process, such risks are minimized. Subjects will be informed that they are participating in an experimental study to determine the effectiveness of acupuncture versus massage for chronic pain. They have the chance to be randomized to either the acupuncture or the massage group. At the end of the study, subjects will be offered the opportunity to discuss the findings with the PI. Additionally, some of the questions in the questionnaire may elicit distress among subjects. If a subject demonstrates clinically significant distress, s/he will be referred to the appropriate clinical and psychosocial services at MSK. Dr. Mao, the PI, has extensive clinical experience in treating physical and psychological distress. During the study period, if the research staff identifies any patients who are psychologically distressed, they will notify Drs. Mao or Deng immediately to facilitate appropriate evaluation and treatment.

Financial and Legal Risks: There are no financial or legal risks to the study participants. All research interventions and evaluations are provided free of charge to study participants.

Privacy and/or Confidentiality Risks: There is a small risk of loss of privacy or confidentiality as someone could get access to the personal information in the study participants' study records.

15.1 Serious Adverse Event (SAE) Reporting

An adverse event is considered serious if it results in ANY of the following outcomes:

- Death
- A life-threatening adverse event
- An adverse event that results in inpatient hospitalization or prolongation of existing hospitalization
- A persistent or significant incapacity or substantial disruption of the ability to conduct normal life functions
- A congenital anomaly/birth defect
- Important Medical Events (IME) that may not result in death, be life threatening, or require hospitalization may be considered serious when, based upon medical judgment, they may jeopardize the patient or participant and may require medical or surgical intervention to prevent one of the outcomes listed in this definition

Note: Hospital admission for a planned procedure/disease treatment is not considered an SAE.

SAE reporting is required as soon as the participant starts investigational treatment/intervention. SAE reporting will include all SAEs that are related to the intervention. SAEs that are unrelated to the protocol intervention will not be reported. If a participant does experience an SAE related to intervention, reporting guidelines will be followed. SAE reporting is required for 30-days after the participant's last investigational

treatment/intervention. Any event that occur after the 30-day period that is unexpected and at least possibly related to protocol treatment must be reported.

Please note: Any SAE that occurs prior to the start of investigational treatment/intervention and is related to a screening test or procedure (i.e., a screening biopsy) must be reported.

All SAEs must be submitted in PIMS. If an SAE requires submission to the HRPP office per IRB SOP RR-408 'Reporting of Serious Adverse Events', the SAE report must be submitted within 5 calendar days of the event. All other SAEs must be submitted within 30 calendar days of the event.

The report should contain the following information:

- The date the adverse event occurred
- The adverse event
- The grade of the event
- Relationship of the adverse event to the treatment(s)
- If the AE was expected
- Detailed text that includes the following
    - A explanation of how the AE was handled
    - A description of the participant's condition
    - Indication if the participant remains on the study
- If an amendment will need to be made to the protocol and/or consent form
- If the SAE is an Unanticipated Problem

15.2. External SAE Reporting

Not applicable.

16.1 PROTECTION OF HUMAN PARTICIPANTS

Risks/Benefits Assessment: Although the risks associated with participation in the proposed study are minimal, all potential risks that might occur as a result of participation will be detailed in an informed consent form, and will also be fully discussed with each subject prior to enrollment. We will also explain to each subject that in the unlikely event of physical injury directly resulting from the research procedures, every effort will be made to make available the facilities and professional skills of MSK. While some risks are unpredictable, every precaution consistent with the best medical practices to protect the health and safety of study participants will be taken. We will document all AEs and report any SAEs promptly to the IRB. The principal investigator and co-investigators will be responsible for judging the nature, severity, and attribution of any adverse events.

Protection Against Acupuncture Risks. The risks associated with acupuncture are minor, and there are very few serious side effects. The most common side effects are mild pain on insertion of the needle. There is a possibility of a small amount of bleeding or bruising. Sometimes, pain in joints and muscles may get worse with acupuncture shortly after treatment. A licensed acupuncturist will administer the acupuncture. Every effort will be made to ensure the safety and comfort of the study participants, including wiping the needling site

with alcohol before the procedure and wiping the needling site with sterile gauze. Adverse events will be recorded during each clinical visit. If participants report any serious side effects, the acupuncturist will inform the PI immediately to address the safety issue. Any SAEs will be reported to the IRB.

Protection Against Massage Risks. The risks associated with massage are minor. A licensed massage therapist will deliver the massage. The most commonly reported side effects are localized temporary fatigue and soreness. Every effort will be made to ensure the safety and comfort of the study participants. Adverse events will be recorded during each clinical visit. If participants report any serious side effects, the massage therapist will inform the PI immediately to address the safety issue. Any SAEs will be reported to the IRB.

Protection Against Psychological Distress Risks. Throughout the study period, if the research staff identifies any subjects who are psychologically distressed, they will notify Drs. Mao or Deng immediately to facilitate appropriate evaluation and treatment. If a subject demonstrates clinically significant distress, s/he will be referred to the appropriate clinical and psychosocial services at MSK.

Financial and Legal Risks: All research interventions and evaluations are provided free of charge to study participants. To encourage completion of all study procedures, participants will receive $40 for completing the Week 10 assessment and $60 for completing the Week 26 assessment (total of $100 per participant).

Protection Against Privacy and/or Confidentiality Risks. Information about study participants will be kept confidential and managed according to HIPAA requirements. MSK's Privacy Office may allow the use and disclosure of protected health information pursuant to a completed and signed Research Authorization form. The use and disclosure of protected health information will be limited to the individuals described in the Research Authorization form. Confidentiality of the participants will be maintained through de-identification processes. We will protect all identifiable information by removing it from our data and assigning each participant a unique study ID. The log linking our study ID and original data source (including name and MRN) will be kept in a separate and password-protected database. The paper data files will be kept in locked cabinets and electronic files will be kept in password-protected databases. The patient's name or any other personally identifying information will not be used in reports or publications resulting from the study. Only authorized representatives of MSK, the Food and Drug Administration, or other authorized agencies may inspect the patient's records.

Risk management and emergency response: At each study visit, the acupuncturist, massage therapist and/or CRC will ask the patients if they have experienced any adverse events (AEs) during the past week. All related AEs will be recorded in an AE log which includes the date of onset and cessation of the AE, severity of AE (i.e. mild, moderate, severe), and relationship to study intervention (i.e. none, possible, probable, definite). The PI (Dr. Mao) or Medical Director (Dr. Deng) will review all recorded AEs in a timely manner. Additionally, all patients will be instructed to contact the CRC immediately if they experience any troubling side effects or worsening of symptoms, or have emergency room care or hospitalizations. Patients will be instructed to return to the clinic for an unscheduled study visit for further evaluation and treatment (if clinically warranted). Any patient who experiences an AE that, in the opinion of the PI, would warrant discontinuing treatment, will be discontinued from the trial. Given this

level of safety monitoring, we anticipate that potentially dangerous AEs resulting from the study intervention will be detected and treated in a timely manner. We will follow up on all related AEs until they have been resolved. All SAEs, regardless of whether or not they are unrelated to the study treatment, will be reported to the IRB.

Potential Benefits: Study participants may experience an improvement in their pain and/or other cancer-related co-morbidities (e.g. sleep problems and fatigue). Improving pain and other problematic symptoms often leads to an improvement in overall physical and emotional well-being. However, acupuncture or massage may or may not be effective for any given patient. This research can help the medical community understand which treatment is more effective for managing pain and therefore has the potential to provide the most definitive findings than previous studies to inform patient and provider decision-making about pain management in the context of the advanced cancer population. The risks to study participants are small in comparison to the potential benefit to patients with advanced cancer that will result from the conduct of this study.

Alternative to Participation: The alternative to participating in the study is to receive standard pain management care without acupuncture or massage or receive these therapies outside of this study. During the informed consent process, potential study participants will be informed of this alternative, that their participation in the study is entirely voluntary, and that their care will not be affected in any way if they decide not to participate in the study.

Risk/Benefit Ratio: The potential benefits of this study far outweigh the potential risks. Chronic pain is a common and debilitating symptom that is experienced by many individuals with advanced cancer. The results of the proposed study will have an immediate impact to help advanced cancer patients suffering from chronic pain make informed and evidence-based decisions about how to most effectively address chronic musculoskeletal pain and co-occurring symptoms. Thus, this study has the potential to improve symptom burden and wellbeing for thousands of individuals whose life is impacted by chronic pain. This research also has the potential to generalize to other chronic conditions and the population at large. We will carefully monitor any adverse events related to acupuncture or massage, and minimize the risks for research subjects.

16.1 Privacy

MSK's Privacy Office may allow the use and disclosure of protected health information pursuant to a completed and signed Research Authorization form. The use and disclosure of protected health information will be limited to the individuals/entities described in the Research Authorization form. A Research Authorization form must be approved by the IRB and Privacy Board (IRB/PB).

The consent indicates that individualized de-identified information collected for the purposes of this study may be shared with other qualified researchers. Only researchers who have received approval from MSK will be allowed to access this information which will not include protected health information, such as the participant's name, except for dates. It is also stated in the Research Authorization that their research data may be shared with others at the time of study publication.

16.2 Data Management

The CRC(s) assigned to this study will be responsible for project compliance, data collection, abstraction and entry, data reporting, regulatory and quality control monitoring, problem identification, and prioritization. Coordination of the study team activities will be the responsibility of our Clinical Research Supervisor (CRS) and/or Clinical Research Manager (CRM). The CRS and CRM will work with the CRC on problem resolution, organization, and quality control. We hold regular meetings attended by the research staff and the Principal Investigator to review study progress and to manage any difficulties encountered. For any communication with participants, all security precautions will be taken, including making sure to activate MSKSecure in e-mail correspondences.

The data collected for this study will be entered into either CRDB, Excel, Access or REDCap secure study databases based on the database functionality. A minimal dataset will be entered into CRDB, and a study tracker will be in Excel. Participants will be asked to complete patient reported outcomes assessments online using REDCap, as described below. If they prefer, patients will have the option to complete the measures via pencil and paper on scannable forms or over the phone with a CRC to reduce participant burden and ensure timely completion.

REDCap (Research Electronic Data Capture) is a data management software system supported by the Clinical Research Administration (CRA) at MSK. Members of the CRA supporting the REDCap software will have access to REDCap projects hosted by MSK's servers for the purpose of ensuring the proper functioning of the database and the overall software system. REDCap is a tool for the creation of customized, secure data management systems including web-based data entry forms, reporting tools, and a full array of security features including user- and group-based privileges with a full audit trail of data manipulation and export procedures. REDCap is maintained on MSK-owned servers that are kept in a locked server room with appropriate environmental modifications (e.g. proper ventilation, power redundancy and fault tolerance arrangement) and backed up nightly with some back-up tapes stored off-site. The MSK Information Systems group is responsible for applying all operating system patches and security updates to the REDCap servers. All connections to REDCap utilize encrypted (SSL-based) connections. Nationally, the REDCap software is developed, enhanced, and supported through a multi-institutional consortium led by Vanderbilt University.

Source documentation will be available to support the computerized patient data. The confidentiality of patient information will be carefully protected. Following data entry by Integrative Medicine Service research staff, data will be maintained in a secure location in the Integrative Medicine offices. All data will be stored in a fashion consistent with FDA guidelines (21CFR11 compliant) and HIPAA security rules.

Final data sets for publication will be locked and stored centrally for potential future access requests from outside entities.

16.3 Quality Assurance

Weekly registration reports will be generated to monitor patient accruals and completeness of registration data. Routine data quality reports will be generated to assess missing data and inconsistencies. Accrual rates and extent and accuracy of evaluations and follow-up will be monitored periodically throughout the study period and potential problems will be brought to the attention of the study team for discussion and action. Random-sample data quality and protocol compliance audits will be conducted by the study team, at a minimum of two times per year, more frequently if indicated.

16.4 Data and Safety Monitoring

The Data and Safety Monitoring (DSM) Plans at Memorial Sloan Kettering were approved by the National Cancer Institute in August 2018. The plans address the new policies set forth by the NCI in the document entitled "Policy of the National Cancer Institute for Data and Safety Monitoring of Clinical Trials."

There are several different mechanisms by which clinical studies are monitored for data, safety and quality. At a departmental/PI level there exists procedures for quality control by the research team(s). Institutional processes in place for quality assurance include protocol monitoring, compliance and data verification audits, staff education on clinical research QA and two institutional committees that are responsible for monitoring the activities of our clinical trials programs. The committees: *Data and Safety Monitoring Committee (DSMC)* for Phase I and II clinical trials, and the *Data and Safety Monitoring Board (DSMB)* for Phase III clinical trials, report to the Deputy Physician-in-Chief, Clinical Research.

During the protocol development and review process, each protocol will be assessed for its level of risk and degree of monitoring required.

The MSK DSMB monitors phase III trials and the DSMC monitors non-phase III trials. The DSMB/C have oversight over the following trials:

- MSK Investigator Initiated Trials (IITs; MSK as sponsor)
- External studies where MSK is the data coordinating center
- Low risk studies identified as requiring DSMB/C review

The DSMC will initiate review following the enrollment of the first participant/or by the end of the year one if no accruals and will continue for the study lifecycle until there are no participants under active therapy and the protocol has closed to accrual. The DSMB will initiate review once the protocol is open to accrual.

17.1 REFERENCES

1. Dowell D, Haegerich TM, Chou R. CDC Guideline for Prescribing Opioids for Chronic Pain--United States, 2016. *Jama.* 2016;315(15):1624-1645.

2. Commission TJ. R3 Report Issue 11: Pain assessment and management standards for hospitals 2018; https://www.jointcommission.org/r3_issue_11/. Accessed June 25, 2018.
3. Paice JA, Portenoy R, Lacchetti C, et al. Management of Chronic Pain in Survivors of Adult Cancers: American Society of Clinical Oncology Clinical Practice Guideline. *Journal of clinical oncology : official journal of the American Society of Clinical Oncology.* 2016;34(27):3325-3345.
4. Swarm RA, Abernethy AP, Anghelescu DL, et al. Adult cancer pain. *Journal of the National Comprehensive Cancer Network : JNCCN.* 2013;11(8):992-1022.
5. ACS. Cancer Facts and Statistics. *American Cancer Society.* 2017.
6. Miller KD, Siegel RL, Lin CC, et al. Cancer treatment and survivorship statistics, 2016. *CA Cancer J Clin.* 2016;66(4):271-289.
7. Delgado-Guay MO, Ferrer J, Ochoa J, et al. Characteristics and Outcomes of Advanced Cancer Patients Who Received Palliative Care at a Public Hospital Compared with Those at a Comprehensive Cancer Center. *Journal of palliative medicine.* 2018;21(5):678-685.
8. Cleeland CS, Zhao F, Chang VT, et al. The symptom burden of cancer: Evidence for a core set of cancer-related and treatment-related symptoms from the Eastern Cooperative Oncology Group Symptom Outcomes and Practice Patterns study. *Cancer.* 2013;119(24):4333-4340.
9. Johnsen AT, Petersen MA, Pedersen L, Groenvold M. Symptoms and problems in a nationally representative sample of advanced cancer patients. *Palliative medicine.* 2009;23(6):491-501.
10. Kirkova J, Rybicki L, Walsh D, Aktas A, Davis MP, Karafa MT. The relationship between symptom prevalence and severity and cancer primary site in 796 patients with advanced cancer. *The American journal of hospice & palliative care.* 2011;28(5):350-355.
11. van den Beuken-van Everdingen MH, Hochstenbach LM, Joosten EA, Tjan-Heijnen VC, Janssen DJ. Update on Prevalence of Pain in Patients With Cancer: Systematic Review and Meta-Analysis. *Journal of pain and symptom management.* 2016;51(6):1070-1090 e1079.
12. Porta-Sales J, Nabal-Vicuna M, Vallano A, et al. Have We Improved Pain Control in Cancer Patients? A Multicenter Study of Ambulatory and Hospitalized Cancer Patients. *Journal of palliative medicine.* 2015;18(11):923-932.
13. Berger AM, Mooney K, Alvarez-Perez A, et al. Cancer-Related Fatigue, Version 2.2015. *Journal of the National Comprehensive Cancer Network : JNCCN.* 2015;13(8):1012-1039.
14. Bower JE, Bak K, Berger A, et al. Screening, assessment, and management of fatigue in adult survivors of cancer: an American Society of Clinical oncology clinical practice guideline adaptation. *Journal of clinical oncology : official journal of the American Society of Clinical Oncology.* 2014;32(17):1840-1850.
15. Reilly CM, Bruner DW, Mitchell SA, et al. A literature synthesis of symptom prevalence and severity in persons receiving active cancer treatment. *Supportive care in cancer : official journal of the Multinational Association of Supportive Care in Cancer.* 2013;21(6):1525-1550.
16. Stark L, Tofthagen C, Visovsky C, McMillan SC. The Symptom Experience of Patients with Cancer. *Journal of hospice and palliative nursing : JHPN : the official journal of the Hospice and Palliative Nurses Association.* 2012;14(1):61-70.

17. Sharma N, Hansen CH, O'Connor M, et al. Sleep problems in cancer patients: prevalence and association with distress and pain. *Psycho-oncology.* 2012;21(9):1003-1009.
18. Mao H, Bao T, Shen X, et al. Prevalence and risk factors for fatigue among breast cancer survivors on aromatase inhibitors. *European journal of cancer (Oxford, England : 1990).* 2018;101:47-54.
19. Dong ST, Butow PN, Costa DS, Lovell MR, Agar M. Symptom clusters in patients with advanced cancer: a systematic review of observational studies. *Journal of pain and symptom management.* 2014;48(3):411-450.
20. Dong ST, Butow PN, Tong A, et al. Patients' experiences and perspectives of multiple concurrent symptoms in advanced cancer: a semi-structured interview study. *Supportive care in cancer : official journal of the Multinational Association of Supportive Care in Cancer.* 2016;24(3):1373-1386.
21. Walsh D, Donnelly S, Rybicki L. The symptoms of advanced cancer: relationship to age, gender, and performance status in 1,000 patients. *Supportive care in cancer : official journal of the Multinational Association of Supportive Care in Cancer.* 2000;8(3):175-179.
22. Walsh D, Rybicki L. Symptom clustering in advanced cancer. *Supportive care in cancer : official journal of the Multinational Association of Supportive Care in Cancer.* 2006;14(8):831-836.
23. Dodd MJ, Miaskowski C, Paul SM. Symptom clusters and their effect on the functional status of patients with cancer. *Oncology nursing forum.* 2001;28(3):465-470.
24. Tick H, Nielsen A, Pelletier KR, et al. Evidence-Based Nonpharmacologic Strategies for Comprehensive Pain Care: The Consortium Pain Task Force White Paper. *Explore (New York, NY).* 2018;14(3):177-211.
25. Zylla D, Steele G, Shapiro A, Richter S, Gupta P. Impact of opioid use on health care utilization and survival in patients with newly diagnosed stage IV malignancies. *Supportive care in cancer : official journal of the Multinational Association of Supportive Care in Cancer.* 2018;26(7):2259-2266.
26. Mao JJ, Kapur R. Acupuncture in primary care. *Prim Care.* 2010;37(1):105-117.
27. White A. A cumulative review of the range and incidence of significant adverse events associated with acupuncture. *Acupunct Med.* 2004;22(3):122-133.
28. Deng G, Bao T, Mao JJ. Understanding the Benefits of Acupuncture Treatment for Cancer Pain Management. *Oncology (Williston Park, NY).* 2018;32(6):310-316.
29. MacPherson H, Vertosick EA, Foster NE, et al. The persistence of the effects of acupuncture after a course of treatment: a meta-analysis of patients with chronic pain. *Pain.* 2017;158(5):784-793.
30. Vickers AJ, Cronin AM, Maschino AC, et al. Acupuncture for chronic pain: individual patient data meta-analysis. *Arch Intern Med.* 2012;172(19):1444-1453.
31. Vickers AJ, Vertosick EA, Lewith G, et al. Acupuncture for Chronic Pain: Update of an Individual Patient Data Meta-Analysis. *J Pain.* 2018;19(5):455-474.
32. Chiu HY, Hsieh YJ, Tsai PS. Systematic review and meta-analysis of acupuncture to reduce cancer-related pain. *European journal of cancer care.* 2017;26(2).
33. Hershman DL, Unger JM, Greenlee H, et al. Effect of Acupuncture vs Sham Acupuncture or Waitlist Control on Joint Pain Related to Aromatase Inhibitors Among Women With Early-Stage Breast Cancer: A Randomized Clinical Trial. *Jama.* 2018;320(2):167-176.

34. Mao JJ, Farrar JT, Bruner D, et al. Electroacupuncture for fatigue, sleep, and psychological distress in breast cancer patients with aromatase inhibitor-related arthralgia: a randomized trial. *Cancer.* 2014;120(23):3744-3751.
35. Boyd C, Crawford C, Paat CF, et al. The Impact of Massage Therapy on Function in Pain Populations-A Systematic Review and Meta-Analysis of Randomized Controlled Trials: Part II, Cancer Pain Populations. *Pain medicine (Malden, Mass).* 2016;17(8):1553-1568.
36. Buckenmaier C, Cambron J, Werner R, et al. Massage Therapy for Pain—Call to Action. *Pain medicine (Malden, Mass).* 2016;17(7):1211-1214.
37. Mao JJ, Wagner KE, Seluzicki CM, et al. Integrating Oncology Massage Into Chemoinfusion Suites: A Program Evaluation. *J Oncol Pract.* 2017;13(3):e207-e216.
38. Crawford C, Boyd C, Paat CF, et al. The Impact of Massage Therapy on Function in Pain Populations-A Systematic Review and Meta-Analysis of Randomized Controlled Trials: Part I, Patients Experiencing Pain in the General Population. *Pain medicine (Malden, Mass).* 2016;17(7):1353-1375.
39. Cherkin DC, Sherman KJ, Kahn J, et al. A comparison of the effects of 2 types of massage and usual care on chronic low back pain: a randomized, controlled trial. *Annals of internal medicine.* 2011;155(1):1-9.
40. Perlman A, Fogerite SG, Glass O, et al. Efficacy and Safety of Massage for Osteoarthritis of the Knee: a Randomized Clinical Trial. *Journal of general internal medicine.* 2019;34(3):379-386.
41. Kutner JS, Smith MC, Corbin L, et al. Massage therapy versus simple touch to improve pain and mood in patients with advanced cancer: a randomized trial. *Annals of internal medicine.* 2008;149(6):369-379.
42. Lopez G, Liu W, Milbury K, et al. The effects of oncology massage on symptom self-report for cancer patients and their caregivers. *Supportive care in cancer : official journal of the Multinational Association of Supportive Care in Cancer.* 2017;25(12):3645-3650.
43. Cassileth BR, Vickers AJ. Massage therapy for symptom control: outcome study at a major cancer center. *Journal of pain and symptom management.* 2004;28(3):244-249.
44. Vickers AJ, Linde K. Acupuncture for chronic pain. *Jama.* 2014;311(9):955-956.
45. Chou R, Deyo R, Friedly J, et al. Nonpharmacologic Therapies for Low Back Pain: A Systematic Review for an American College of Physicians Clinical Practice Guideline. *Annals of internal medicine.* 2017;166(7):493-505.
46. Lee SH, Kim JY, Yeo S, Kim SH, Lim S. Meta-Analysis of Massage Therapy on Cancer Pain. *Integrative cancer therapies.* 2015;14(4):297-304.
47. Qaseem A, Wilt TJ, McLean RM, Forciea MA, Clinical Guidelines Committee of the American College of P. Noninvasive Treatments for Acute, Subacute, and Chronic Low Back Pain: A Clinical Practice Guideline From the American College of Physicians. *Annals of internal medicine.* 2017;166(7):514-530.
48. Network NCC. NCCN Clinical Practice Guidelines in Oncology: Adult Cancer Pain Version I.2018. 2018; https://www.nccn.org/professionals/physician_gls/pdf/pain.pdf. Accessed June 22, 2018.
49. Yun H, Sun L, Mao JJ. Growth of Integrative Medicine at Leading Cancer Centers Between 2009 and 2016: A Systematic Analysis of NCI-Designated Comprehensive Cancer Center Websites. *Journal of the National Cancer Institute Monographs.* 2017;2017(52).

50. Heymach J, Krilov L, Alberg A, et al. Clinical Cancer Advances 2018: Annual Report on Progress Against Cancer From the American Society of Clinical Oncology. *Journal of clinical oncology : official journal of the American Society of Clinical Oncology.* 2018;36(10):1020-1044.
51. Zia FZ, Olaku O, Bao T, et al. The National Cancer Institute's Conference on Acupuncture for Symptom Management in Oncology: State of the Science, Evidence, and Research Gaps. *Journal of the National Cancer Institute Monographs.* 2017;2017(52).
52. Laird BJ, Scott AC, Colvin LA, et al. Pain, depression, and fatigue as a symptom cluster in advanced cancer. *Journal of pain and symptom management.* 2011;42(1):1-11.
53. Ganesh V, Zhang L, Wan BA, et al. Symptom clusters using the EORTC QLQ-C15-PAL in palliative radiotherapy. *Annals of palliative medicine.* 2018;7(2):192-204.
54. Dodd MJ, Cho MH, Cooper BA, Miaskowski C. The effect of symptom clusters on functional status and quality of life in women with breast cancer. *European journal of oncology nursing : the official journal of European Oncology Nursing Society.* 2010;14(2):101-110.
55. Miaskowski C, Cooper BA, Paul SM, et al. Subgroups of patients with cancer with different symptom experiences and quality-of-life outcomes: a cluster analysis. *Oncology nursing forum.* 2006;33(5):E79-89.
56. Mao JJ, Palmer CS, Healy KE, Desai K, Amsterdam J. Complementary and alternative medicine use among cancer survivors: a population-based study. *Journal of cancer survivorship : research and practice.* 2011;5(1):8-17.
57. Garland SN, Eriksen W, Song S, et al. Factors that shape preference for acupuncture or cognitive behavioral therapy for the treatment of insomnia in cancer patients. *Supportive care in cancer : official journal of the Multinational Association of Supportive Care in Cancer.* 2018;26(7):2407-2415.
58. Mao JJ, Leed R, Bowman MA, et al. Acupuncture for hot flashes: decision making by breast cancer survivors. *Journal of the American Board of Family Medicine : JABFM.* 2012;25(3):323-332.
59. Latte-Naor S, Sidlow R, Sun L, Li QS, Mao JJ. Influence of family on expected benefits of complementary and alternative medicine (CAM) in cancer patients. *Supportive care in cancer : official journal of the Multinational Association of Supportive Care in Cancer.* 2018;26(6):2063-2069.
60. Sun L, Yang Y, Vertosick E, Jo S, Sun G, Mao JJ. Do Perceived Needs Affect Willingness to Use Traditional Chinese Medicine for Survivorship Care Among Chinese Cancer Survivors? A Cross-Sectional Survey. *Journal of global oncology.* 2017;3(6):692-700.
61. Mao JJ, Palmer SC, Straton JB, et al. Cancer survivors with unmet needs were more likely to use complementary and alternative medicine. *Journal of cancer survivorship : research and practice.* 2008;2(2):116-124.
62. Romero SAD, Jones L, Bauml JM, Li QS, Cohen RB, Mao JJ. The association between fatigue and pain symptoms and decreased physical activity after cancer. *Supportive care in cancer : official journal of the Multinational Association of Supportive Care in Cancer.* 2018.
63. Mao JJ, Xie SX, Farrar JT, et al. A randomised trial of electro-acupuncture for arthralgia related to aromatase inhibitor use. *European journal of cancer (Oxford, England : 1990).* 2014;50(2):267-276.

64. Bauml J, Xie SX, Farrar JT, et al. Expectancy in real and sham electroacupuncture: does believing make it so? *Journal of the National Cancer Institute Monographs.* 2014;2014(50):302-307.
65. Cherkin DC, Eisenberg D, Sherman KJ, et al. Randomized trial comparing traditional Chinese medical acupuncture, therapeutic massage, and self-care education for chronic low back pain. *Arch Intern Med.* 2001;161(8):1081-1088.
66. Cheng X, ed *Chinese acupuncture and moxibustion.* Beijing, China: Foreign Languages Press; 1987.
67. Mao JJ, Farrar JT, Armstrong K, Donahue A, Ngo J, Bowman MA. De qi: Chinese acupuncture patients' experiences and beliefs regarding acupuncture needling sensation--an exploratory survey. *Acupunct Med.* 2007;25(4):158-165.
68. Helms J. *Acupuncture Energetics: A Clinical Approach for Physicians.* First ed: Medical Acupuncture Publishers; 1995.
69. Han JS. Acupuncture: neuropeptide release produced by electrical stimulation of different frequencies. *Trends Neurosci.* 2003;26(1):17-22.
70. Han JS. Acupuncture and endorphins. *Neurosci Lett.* 2004;361(1-3):258-261.
71. Berman BM, Lao L, Langenberg P, Lee WL, Gilpin AM, Hochberg MC. Effectiveness of acupuncture as adjunctive therapy in osteoarthritis of the knee: a randomized, controlled trial. *Annals of internal medicine.* 2004;141(12):901-910.
72. Vas J, Mendez C, Perea-Milla E, et al. Acupuncture as a complementary therapy to the pharmacological treatment of osteoarthritis of the knee: randomised controlled trial. *BMJ.* 2004;329(7476):1216.
73. Mao JJ, Bruner DW, Stricker C, et al. Feasibility trial of electroacupuncture for aromatase inhibitor--related arthralgia in breast cancer survivors. *Integrative cancer therapies.* 2009;8(2):123-129.
74. MacDonald G. *Medicine hands: massage therapy for people with cancer.* Simon and Schuster; 2014.
75. Walton T. *Medical conditions and massage therapy: a decision tree approach.* Lippincott Williams & Wilkins; 2010.
76. Chen LX, Mao JJ, Fernandes S, et al. Integrating acupuncture with exercise-based physical therapy for knee osteoarthritis: a randomized controlled trial. *J Clin Rheumatol.* 2013;19(6):308-316.
77. Rodin G, Lo C, Rydall A, et al. Managing Cancer and Living Meaningfully (CALM): A Randomized Controlled Trial of a Psychological Intervention for Patients With Advanced Cancer. *Journal of Clinical Oncology.* 2018;36(23):2422-2432.
78. Lo C, Zimmermann C, Rydall A, et al. Longitudinal study of depressive symptoms in patients with metastatic gastrointestinal and lung cancer. *Journal of clinical oncology : official journal of the American Society of Clinical Oncology.* 2010;28(18):3084-3089.
79. Robinson-Papp J, George MC, Wongmek A, et al. The Quantitative Analgesic Questionnaire: A Tool to Capture Patient-Reported Chronic Pain Medication Use. *Journal of pain and symptom management.* 2015;50(3):381-386.
80. Garland SN, Xie SX, Li Q, Seluzicki C, Basal C, Mao JJ. Comparative effectiveness of electro-acupuncture versus gabapentin for sleep disturbances in breast cancer survivors with hot flashes: a randomized trial. *Menopause (New York, NY).* 2017;24(5):517-523.
81. Mao JJ, Bowman MA, Xie SX, Bruner D, DeMichele A, Farrar JT. Electroacupuncture Versus Gabapentin for Hot Flashes Among Breast Cancer Survivors: A Randomized Placebo-Controlled Trial. *Journal of clinical oncology : official journal of the American Society of Clinical Oncology.* 2015;33(31):3615-3620.

82. Cleeland CS, Ryan KM. Pain assessment: global use of the Brief Pain Inventory. *Ann Acad Med Singapore.* 1994;23(2):129-138.
83. Farrar JT, Portenoy RK, Berlin JA, Kinman JL, Strom BL. Defining the clinically important difference in pain outcome measures. *Pain.* 2000;88(3):287-294.
84. Farrar JT, Young JP, Jr., LaMoreaux L, Werth JL, Poole RM. Clinical importance of changes in chronic pain intensity measured on an 11-point numerical pain rating scale. *Pain.* 2001;94(2):149-158.
85. Farrar JT, Pritchett YL, Robinson M, Prakash A, Chappell A. The clinical importance of changes in the 0 to 10 numeric rating scale for worst, least, and average pain intensity: analyses of data from clinical trials of duloxetine in pain disorders. *J Pain.* 2010;11(2):109-118.
86. Hurst H, Bolton J. Assessing the clinical significance of change scores recorded on subjective outcome measures. *Journal of manipulative and physiological therapeutics.* 2004;27(1):26-35.
87. Mendoza TR, Wang XS, Cleeland CS, et al. The rapid assessment of fatigue severity in cancer patients: use of the Brief Fatigue Inventory. *Cancer.* 1999;85(5):1186-1196.
88. Lin CC, Chang AP, Chen ML, Cleeland CS, Mendoza TR, Wang XS. Validation of the Taiwanese version of the Brief Fatigue Inventory. *Journal of pain and symptom management.* 2006;32(1):52-59.
89. Bastien CH, Vallieres A, Morin CM. Validation of the Insomnia Severity Index as an outcome measure for insomnia research. *Sleep medicine.* 2001;2(4):297-307.
90. Savard MH, Savard J, Simard S, Ivers H. Empirical validation of the Insomnia Severity Index in cancer patients. *Psycho-oncology.* 2005;14(6):429-441.
91. Morin CM, Belleville G, Belanger L, Ivers H. The Insomnia Severity Index: psychometric indicators to detect insomnia cases and evaluate treatment response. *Sleep.* 2011;34(5):601-608.
92. Zigmond AS, Snaith RP. The hospital anxiety and depression scale. *Acta Psychiatr Scand.* 1983;67(6):361-370.
93. Moorey S, Greer S, Watson M, et al. The factor structure and factor stability of the hospital anxiety and depression scale in patients with cancer. *Br J Psychiatry.* 1991;158:255-259.
94. Smith AB, Selby PJ, Velikova G, et al. Factor analysis of the Hospital Anxiety and Depression Scale from a large cancer population. *Psychol Psychother.* 2002;75(Pt 2):165-176.
95. Hays RD, Bjorner JB, Revicki DA, Spritzer KL, Cella D. Development of physical and mental health summary scores from the patient-reported outcomes measurement information system (PROMIS) global items. *Qual Life Res.* 2009;18(7):873-880.
96. Revicki DA, Kawata AK, Harnam N, Chen WH, Hays RD, Cella D. Predicting EuroQol (EQ-5D) scores from the patient-reported outcomes measurement information system (PROMIS) global items and domain item banks in a United States sample. *Qual Life Res.* 2009;18(6):783-791.
97. Rothrock NE, Hays RD, Spritzer K, Yount SE, Riley W, Cella D. Relative to the general US population, chronic diseases are associated with poorer health-related quality of life as measured by the Patient-Reported Outcomes Measurement Information System (PROMIS). *J Clin Epidemiol.* 2010;63(11):1195-1204.
98. Barile JP, Reeve BB, Smith AW, et al. Monitoring population health for Healthy People 2020: evaluation of the NIH PROMIS(R) Global Health, CDC Healthy Days, and satisfaction with life instruments. *Qual Life Res.* 2013;22(6):1201-1211.

99. Mao JJ, Armstrong K, Farrar JT, Bowman MA. Acupuncture expectancy scale: development and preliminary validation in China. *Explore (New York, NY).* 2007;3(4):372-377.
100. Colagiuri B, Smith CA. A systematic review of the effect of expectancy on treatment responses to acupuncture. *Evidence-based complementary and alternative medicine : eCAM.* 2012;2012:857804.
101. Kalauokalani D, Cherkin DC, Sherman KJ, Koepsell TD, Deyo RA. Lessons from a trial of acupuncture and massage for low back pain: patient expectations and treatment effects. *Spine.* 2001;26(13):1418-1424.
102. Tango T. Power and sample size for the S:T repeated measures design combined with a linear mixed-effects model allowing for missing data. *Journal of biopharmaceutical statistics.* 2017;27(6):963-974.
103. Benjamini Y, Hochberg Y. Controlling the False Discovery Rate: A Practical and Powerful Approach to Multiple Testing. *Journal of the Royal Statistical Society Series B (Methodological).* 1995;57(1):289-300.
104. Benjamini Y, Yekutieli D. The control of the false discovery rate in multiple testing under dependency. *Ann Statist.* 2001;29(4):1165-1188.
105. Kent DM, Rothwell PM, Ioannidis JP, Altman DG, Hayward RA. Assessing and reporting heterogeneity in treatment effects in clinical trials: a proposal. *Trials.* 2010;11:85.
106. Henderson NC, Louis TA, Wang C, Varadhan R. Bayesian analysis of heterogeneous treatment effects for patient-centered outcomes research. *Health Services and Outcomes Research Methodology.* 2016;16(4):213-233.
107. Wang C, Louis TA, Henderson NC, Weiss CO, Varadhan R. beanz: An R Package for Bayesian Analysis of Heterogeneous Treatment Effects with a Graphical User Interface. *2018.* 2018;85(7):31.
108. Imai K, Ratkovic M. Estimating treatment effect heterogeneity in randomized program evaluation. *Ann Appl Stat.* 2013;7(1):443-470.
109. Wager S, Athey S. Estimation and Inference of Heterogeneous Treatment Effects using Random Forests. *Journal of the American Statistical Association.* 2017:1-15.
110. Lu K, Luo X, Chen PY. Sample size estimation for repeated measures analysis in randomized clinical trials with missing data. *The international journal of biostatistics.* 2008;4(1):Article 9.
111. Dworkin RH, Turk DC, Wyrwich KW, et al. Interpreting the clinical importance of treatment outcomes in chronic pain clinical trials: IMMPACT recommendations. *J Pain.* 2008;9(2):105-121.
112. Krebs EE, Gravely A, Nugent S, et al. Effect of Opioid vs Nonopioid Medications on Pain-Related Function in Patients With Chronic Back Pain or Hip or Knee Osteoarthritis Pain: The SPACE Randomized Clinical Trial. *Jama.* 2018;319(9):872-882.
113. Little RJ, D'Agostino R, Cohen ML, et al. The prevention and treatment of missing data in clinical trials. *N Engl J Med.* 2012;367(14):1355-1360.
114. Little RJ, Cohen ML, Dickersin K, et al. The design and conduct of clinical trials to limit missing data. *Stat Med.* 2012;31(28):3433-3443.
115. Siddique J, Harel O, Crespi CM. Addressing Missing Data Mechanism Uncertainty using Multiple-Model Multiple Imputation: Application to a Longitudinal Clinical Trial. *The annals of applied statistics.* 2012;6(4):1814-1837.
116. Ibrahim JG, Molenberghs G. Missing data methods in longitudinal studies: a review. *Test (Madrid, Spain).* 2009;18(1):1-43.

18.0 APPENDICES

Appendix 1: Acupuncture Intervention Protocol

Appendix 2: Massage Intervention Protocol

Appendix 3: Recruitment Letter

Appendix 4: Recruitment Flyer

Appendix 5: Recruitment Rack Card

Appendix 6: Patient-Reported Outcome Measures

Appendix 7: Brief Pain Inventory (aka Pain Diary)

Appendix 8: Pain Medication Diary

Appendix 9: Pain History

Appendix 10: No Contact Letter

Appendix 11: Recruitment Flyer 3

Appendix 12: Recruitment Flyer 2

Appendix 13: Study Information Sheet_After consent
