# Supplementary material for: Acupuncture vs Massage for Pain in Patients Living With Advanced Cancer: The IMPACT Randomized Clinical Trial
Source: JAMA Netw Open. 2023 Nov 14;6(11):e2342482. doi: 10.1001/jamanetworkopen.2023.42482 (PMC10646731; doi:10.1001/jamanetworkopen.2023.42482)
Supplement: Supplement 2. — eMethods. Sensitivity Analyses Adjusting for COVID-19–Related Treatment Interruptions eTable. Estimates of Within-Arm and Between-Arm Differences for BPI Worst Pain, Controlling for COVID-19–Related Treatment Interruptions [file jamanetwopen-e2342482-s002.pdf]

Supplementary Online Content

Epstein AS, Liou KT, Romero SAD, et al. Acupuncture vs massage for pain in patients living with advanced cancer: the IMPACT randomized clinical trial. *JAMA Netw Open*. 2023;11(6):e2342482. doi:10.1001/jamanetworkopen.2023.42482

eMethods. Sensitivity Analyses Adjusting for COVID-19–Related Treatment Interruptions

eTable. Estimates of Within-Arm and Between-Arm Differences for BPI Worst Pain, Controlling for COVID-19–Related Treatment Interruptions

This supplementary material has been provided by the authors to give readers additional information about their work.

© 2023 Epstein AS et al. *JAMA Network Open.*

eMethods: Sensitivity Analyses Adjusting for COVID-19-related Treatment Interruptions

When COVID-19 hit New York City in March 2020, we had to put our interventions on hold between March and July 2020. In consultation with co-investigators, statisticians, patient-partners, and the Patient-Centered Outcomes Research Institute (PCORI, funder), we continued data collection for those participants who were willing to provide the data. In addition, some patients later on had to have treatment interruption due to testing positive for COVID-19 (not allowed to enter health care facility). Overall, 53 (17.8%) of participants had at least one treatment-related interruption due to COVID-19. In order to evaluate whether such treatment interruptions affected our primary outcome at the primary end point, we conducted a sensitivity analysis controlling for whether or not patients had experienced a COVID-19-related treatment interruption in our main linear mixed model for Brief Pain Inventory (BPI) worst pain. The model-estimated means and 95% confidence intervals (CIs) for this COVID-adjusted model are presented in Table 3. Naturally, we did not pre-specify this sensitivity analysis in the study protocol because the COVID-19 pandemic started after the study opened to enrollment. However, due to the extenuating circumstance of conducting a clinical trial as the pandemic unfolded, we felt this sensitivity analysis was warranted.

Adjusting for COVID-19 treatment interruption, the difference in BPI worst pain reduction at week 26 between acupuncture and massage was not significant and almost identical to our primary ITT analyses (0.48, 95% confidence interval [CI] -0.03 to 0.99, Cohen's D 0.31, p=0.063). The patients who experienced a COVID-19-related treatment interruption tended to have slightly non-significantly higher pain scores at each time point (beta = 0.27, p = 0.39).

© 2023 Epstein AS et al. *JAMA Network Open.*

eTable: Estimates of Within-Arm and Between-Arm Differences for BPI Worst Pain, Controlling for COVID-19-related Treatment Interruptions

Week	Arm	Mean (95% CI)	Mean Change (95% CI)	Mean Between-Arm Difference (95% CI)	p-value	Effect Size (95% CI)
0	Acupuncture	6.93 (6.66, 7.20)				
10	Acupuncture	4.79 (4.40, 5.17)	-2.14 (-2.52, -1.77)			
26	Acupuncture	4.40 (4.01, 4.79)	-2.53 (-2.92, -2.15)			
0	Massage	6.93 (6.66, 7.20)				
10	Massage	4.29 (3.90, 4.68)	-2.64 (-3.02, -2.25)	-0.49 (-1.00, 0.02)	0.058	-0.32 (-0.65, 0.01)
26	Massage	3.92 (3.54, 4.30)	-3.02 (-3.39, -2.64)	-0.48 (-0.99, 0.03)	0.063	-0.31 (-0.64, 0.02)

Abbreviation: CI, confidence interval

© 2023 Epstein AS et al. *JAMA Network Open.*
