# Supplementary material for: Acupuncture vs Massage for Pain in Patients Living With Advanced Cancer: The IMPACT Randomized Clinical Trial
Source: JAMA Netw Open. 2023 Nov 14;6(11):e2342482. doi: 10.1001/jamanetworkopen.2023.42482 (PMC10646731; doi:10.1001/jamanetworkopen.2023.42482)
Supplement: Supplement 3. — Data Sharing Statement [file jamanetwopen-e2342482-s003.pdf]

Data Sharing Statement

Epstein. Acupuncture vs Massage for Pain in Patients Living With Advanced Cancer. *JAMA Netw Open*. Published November 14, 2023. doi:10.1001/jamanetworkopen.2023.42482

Data
Data available: Yes
Data types: Deidentified participant data
How to access data: Upon reasonable request to study PI Dr. Mao.
When available: With publication

Supporting Documents
Document types: Statistical/analytic code, Informed consent form
How to access documents: Upon reasonable request to study PI Dr. Mao.
When available: With publication

Additional Information
Who can access the data: Upon reasonable request to study PI Dr. Mao.
Types of analyses: Upon reasonable request to study PI Dr. Mao (for a specified purpose).
Mechanisms of data availability: Upon reasonable request to study PI Dr. Mao.
